# Supplementary material for: Transfer learning guided discovery of efficient perovskite oxide for alkaline water oxidation
Source: Nat Commun. 2024 Jul 26;15:6301. doi: 10.1038/s41467-024-50605-5 (PMC11282268; doi:10.1038/s41467-024-50605-5)
Supplement: Supplementary file 1 — Supplementary Information [file 41467_2024_50605_MOESM1_ESM.pdf]

Supplementary Information

Transfer learning guided discovery of efficient perovskite oxide for alkaline water oxidation

Chang Jiang[1, †], Hongyuan He[2, †], Hongquan Guo[1], Xiaoxin Zhang[1], Qingyang Han[1], Yanhong Weng[3], Xianzhu Fu[3], Yinlong Zhu[4], Ning Yan[5], Xin Tu[2,*], Yifei Sun[1,6,7,*]

[1] College of Energy, Xiamen University, Xiamen, 361005, China.

[2] Department of Electrical Engineering and Electronics, University of Liverpool, Liverpool, L69 3GJ, UK.

[3] Shenzhen Key Laboratory of Energy Electrocatalytic Materials, Guangdong Research Center for Interfacial Engineering of Functional Materials, College of Materials Science and Engineering, Shenzhen University, Shenzhen, 518055, China.

[4] Institute for Frontier Science, Nanjing University of Aeronautics and Astronautics, Nanjing, 210001, China.

[5] School of Physics and Technology, Wuhan University, Wuhan, 430072, China.

[6] State Key Laboratory of Physical Chemistry of Solid Surface, Xiamen University, Xiamen, 361005, China.

[7] Shenzhen Research Institute of Xiamen University, Shenzhen, 518057, China.

✪ These authors contribute equally to this work.

Contents:

Supplementary Notes
Supplementary Figs. S1 to S45
Supplementary Tables S1 to S10
References

Supplementary Notes

Supplementary Note 1. Cation encoding

Considering the representative perovskite oxide formula of $(\mathrm{A'_nA''_{1-n}})(\mathrm{B'_mB''_{1-m}})\mathrm{O_x}$ and the electron neutrality of the material, the number of oxygen atoms can be inferred from the aggregate cation valence, using the formula:

$$x = \frac{\sum \text{cation valence}}{2} \tag{1}$$

Both A-site and B-site features are ascertained through the weighted average of their respective cation properties. To implement cation encoding for each element, the elemental properties are computed as the weighted average of all available cation states. Specifically, if element A' comprises a fraction k in valence state 1 and a fraction $(1-k)$ in valence state 2, the corresponding cation radius for A' can be described by the following equation:

$$R_{A'} = R_{A'^1} \times k + R_{A'^2} \times (1-k) \tag{2}$$

where $\mathrm{R_{A'^1}}$ and $\mathrm{R_{A'^2}}$ represent radius of cation A′ in valence state 1 and 2, respectively. The computed values for cationic features corresponding to each element, further elaborated in subsequent sections, adhere to the same computational framework.

Calculations pertaining to cation properties are conducted using the Python library Pymatgen[1]. The specific details for each feature calculation are delineated below:
Valence for A site and B site.

$$\sum V_A = V_{A'} \times n + V_{A''} \times (1-n) \tag{3}$$

$$\sum V_B = V_{B'} \times m + V_{B''} \times (1-m) \tag{4}$$

Cation radius for A site and B site.

$$\sum R_A = R_{A'} \times n + R_{A''} \times (1-n) \tag{5}$$

$$\sum R_B = R_{B'} \times m + R_{B''} \times (1-m) \tag{6}$$

The electronegativity of cation for A site and B site is sourced from literature[2]. Refer to Table S1-2 for details.

$$\sum \chi_A = \chi_{A'} \times n + \chi_{A''} \times (1-n) \tag{7}$$

$$\sum \chi_B = \chi_{B'} \times m + \chi_{B''} \times (1-m) \tag{8}$$

Ionization energies. The $\mathrm{v^{th}}$ ionization energy is selected for cation with valence v.

$$E_{I_{A'}} = E_{I_{A'}}{}^{V_{A'}th} \tag{9}$$

$$E_{I_{B'}} = E_{I_{B'}}{}^{V_{B'}th} \tag{10}$$

$$\sum E_{I_A} = E_{I_{A'}} \times n + E_{I_{A''}} \times (1-n) \tag{11}$$

$$\sum E_{I_B} = E_{I_{B'}} \times m + E_{I_{B''}} \times (1-m) \tag{12}$$

Weighted radius of oxygen.

$$\sum R_O = R_O/x \tag{13}$$

Tolerance factor (with and without weighted radius of oxygen).

$$\tau = \frac{\sum R_A + \sum R_O}{\sqrt{2} \times (\sum R_B + \sum R_O)} \tag{14}$$

$$\tau' = \frac{\sum R_A + R_O}{\sqrt{2} \times (\sum R_B + R_O)} \tag{15}$$

Octahedral factor (with and without weighted radius of oxygen).

$$\mu = \sum R_B / \sum R_O \tag{16}$$

$$\mu' = \sum R_B / R_O \tag{17}$$

Entropy for A site and B site.

$$S_A = -n \times \log(n) - (1-n) \times \log(1-n) \tag{18}$$

$$S_B = -m \times \log(m) - (1-m) \times \log(1-m) \tag{19}$$

Entropy for the whole formula.

$$S = -\left(\frac{n}{2+x}\right) \times \log\left(\frac{n}{2+x}\right) - \left(\frac{1-n}{2+x}\right) \times \log\left(\frac{1-n}{2+x}\right) - \left(\frac{m}{2+x}\right) \times \log\left(\frac{m}{2+x}\right) - \left(\frac{1-m}{2+x}\right) \times \log\left(\frac{1-m}{2+x}\right) - \left(\frac{x}{2+x}\right) \times \log\left(\frac{x}{2+x}\right) \tag{20}$$

Supplementary Note 2. Training and configuration details for auto-encoder with shortcut connections (AESC)

The data were all preprocessed and normalized using a standard scaler. The standard value z of a sample x is calculated as:

$$z = \frac{x-u}{s} \tag{21}$$

where u is the mean of the training sample and s is the standard deviation.

The PyTorch library is employed to implement an AESE with symmetrical encoder and decoder architectures. The encoder consists of a sequence of three interconnected sequential modules, each incorporating a linear layer, followed by a layer normalization unit [3], and a Rectified Linear Unit (ReLU) layer. This chain of modules is then connected to the embedding space via an additional linear layer. Importantly, all modules subsequent to the initial one, as well as the terminal linear layer, feature a concatenation of the original input values, effectively serving as shortcut connections. The decoder maintains structural symmetry with the encoder, comprising three equivalent sequential modules and a terminal linear layer linked to the output space. Shortcuts are integrated in a manner analogous to the encoder with concatenation of the embedding instead. For optimization, the Adam optimizer [4] is employed with a learning rate of $5\text{x}10^{-4}$. The model is trained using a batch size of one and a maximum of 1,000 epochs, with an early termination criterion triggered if no improvement in the optimal metric is recorded after 100 epochs.

The AESC was rigorously optimized using an n-fold cross-validation strategy. The final architecture employed an ensemble technique, averaging the outputs of individual sub-models derived from each cross-validation run, thereby enhancing the overall fitness. Multiple trials were

conducted to ascertain the most effective number of folds and optimal embedding dimensions. Evaluation metrics were anchored on the correlation of determination (R^2) values, specifically comparing the decoded output against the original 20-dimensional cation encodings. Across each cross-validation approach (3, 5, 7, 10, or 15 folds), we explored 10 different parameter sets with neural network neuron numbers from low to high. These architectures differ only in their node counts while retaining their foundational structure. Detailed specifications for these node counts are as follows: [8,8,8], [12,8,8], [12,12,12], [16,12,12], [16,16,16], [24,16,16], [24,24,24], [32,24,24], [32,32,32], and [36,36,36]. These parameter sets represented model simplicity to complexity and were examined for each dimensional configuration from 1 to 8. This cumulative exploration resulted in the examination of 400 unique models, as detailed in Supplementary Fig. 1 for all model results.

Supplementary Note 3. Hyperparameters grid search list for Gradient Boost Regressor [5]

'n_estimators': [3,5,10,20,50]
'learning_rate': [0.01,0.1,0.5]
'subsample': [0.5,0.8,1.0]
'max_depth': [2,3,4,5,6,7,8,9]
'min_samples_split': [2,4,6,8,10]
'min_samples_leaf': [1,2,3,4,5,6,7,8,9]
'max_features': [3,5,7]
'random_state': [1]

A total of 48,600 distinct hyperparameter combinations were explored. For instance, when using K-Means clustering with five clusters and conducting a 3-fold cross-validation, a cumulative total of 729,000 individual GBR models were trained.

Supplementary Note 4. Clustering implementation details

K-Means: clustering with the "lloyd" style[6] is implemented with the Scikit-Learn library. To ensure consistent results across repeated runs, the random state was fixed at 0. The elbow technique is employed to determine the optimal number of clusters (k). This method involves applying k-means clustering to the dataset with varying values of k, ranging from 1 to 20. The objective is to plot the average distance and identify the "elbow" point [7], which represents an inflection point in the rate of decrease of the sum of squared distances (SSD) within each cluster. For each k value, the total SSD is calculated. The "elbow" point serves as a guide for selecting the optimal value of k. Cluster numbers of 5 and 6 are identified as elbow points.

Adjusted Rand Index [7]: The Adjusted Rand Index (ARI) evaluates the similarity between two distinct sets of clusters by examining every possible pair of data points. It determines how many of these pairs are classified similarly or dissimilarly in both the true and predicted groupings The original Rand Index (RI) score is then modified to account for random occurrences, resulting in the ARI score. This is achieved through the following formula:

$$\mathrm{ARI} = \frac{\mathrm{RI} - \mathrm{Anticipated}_{\mathrm{RI}}}{\mathrm{Highest}_{\mathrm{Possible}_{\mathrm{RI}}} - \mathrm{Anticipated}_{\mathrm{RI}}} \tag{22}$$

Classes based on phase-sorting are considered the ground truth (same below).

Completeness [8]: Completeness is evaluated based on the extent to which data points belonging to the same class are grouped into the same clusters.

Homogeneity [8]: Homogeneity is assessed based on the number of clusters that exclusively contain data points from a single class.

V-Measure [8]: V-measure is calculated as the harmonic mean of homogeneity and completeness.

Silhouette Score [9]: The Silhouette Coefficient is determined by averaging two types of distances for each individual data point: the average distance within its own cluster and the average distance to the closest cluster to which it does not belong.

Supplementary Note 5. Global ensembling method

1. For each index i in range(n) where n is number of total data points:
    P_i = []
    For each clustering method:
        For each index j in range(m) where m is number of clusters:
            P_{ij} is the predicted overpotential of GBR trained on cluster j
            D_{ij} is the Euclidean distance between X_i and cluster center C_j
            $P_{ij} = P_{ij} / (1+D_{ij})$
        $P_i = P_i + [P_{i0}, P_{i1}, ..., P_{im}]$
2. Create a new dataset with P_i as input and the true overpotential P_{i_true} corresponding to each data point as output (label).
3. Use Left-one-out to train GBRs that project P_i to P_{i_true}.
4. Combine all GBRs from step 3, using their arithmetic mean of the predictions as the ensembled output result

Supplementary Note 6. Oxide state guessing process

The number of oxygen vacancies significantly influenced the overall valence state distribution of the material. For each compositional formula, ten potential oxygen vacancy levels, ranging from 2.1 to 3.0, were investigated. Based on these compositional and oxygen vacancy parameters, predictive estimations of the valence state distribution were then made.

Notably, while A-site elements are restricted to a specific valence state, multiple valence states are considered for B-site elements, including Mn, Co, Fe, and Ni. To assess the distribution of multiple valence states, we used a minimum unit of 0.01 for the proportions and incorporated specific ratios derived from oxide state distribution proportions reported in the literature.
Valence state estimates were initially based on Pymatgen's 'oxi_state_guesses' function (https://github.com/materialsproject/pymatgen/blob/master/pymatgen/core/composition.py) for the screening process.

To ensure stability and controllability in our valence state prediction process, we developed a localized version of the oxide state guessing function. The results obtained from this localized function showed minor deviations from those generated by Pymatgen, but the disparities were insignificant. For instance, in the first-round prediction of PSCF, the predicted overpotential based

on Pymatgen-generated data points was 365.79 mV ± 18.94 mV, compared to 364.80 mV ± 18.55 mV when using our localized function. We chose to use our local function to recalculate all data points requiring experimental verification. The data presented in the main content is derived from these recalculated data points. Both screening data and recalculated data are presented in the data file. Given that our localized function can accommodate a larger array of possible charge state combinations (e.g., the B-site combinations for CoFeNi could result in a memory exceeding error), we strategically limited our calculations to the multi-charge state distributions of Co and Fe to reduce computational demands.

Novel material prediction begins with generating the compositional formula, specifying the constituent elements and their proportions. In the first-round of predictions, we focused primarily on quaternary and quinary compounds. A combinatorial approach was used to select all possible element combinations from designated A-site and B-site elements. Specifically, the overall cation type is 3 or 4: AA’B or AA’BB’. The A site is fixed to 6 different combinations: Ba+Sr, La+Ca, La+Sr, Pr+Ba, Pr+Ca and Pr+Sr. The B site is selected from Co, Fe, Mn, Nb, Zr, Ni, Cu, Sn, Ir and Ru.

The elemental ratios were systematically varied from 0.1 to 0.9, in increments of 0.2. This approach yielded 7,050 unique compositional formulas. Over 5 million potential valence state distributions were computed in the first round for screening. See Table S3 for first round prediction results of all experimentally evaluated data.

The second round focused on hexahydroxy compounds and employed the same methodology as the first round. This resulted in 9000 unique compositional formulas, with A sites being a fixed combination of Pr+Sr, and B sites being combinations of three elements selected from the first-round prediction. Cumulatively, over 20 million potential valence state distributions were computed in the second round for screening. See Table S4 for second-round prediction results of all experimentally evaluated data.

The third round of predictions serves as the validation phase for the active learning approach and does not introduce any new data points for estimation. See Table S5 for third prediction results of all experimentally evaluated data.

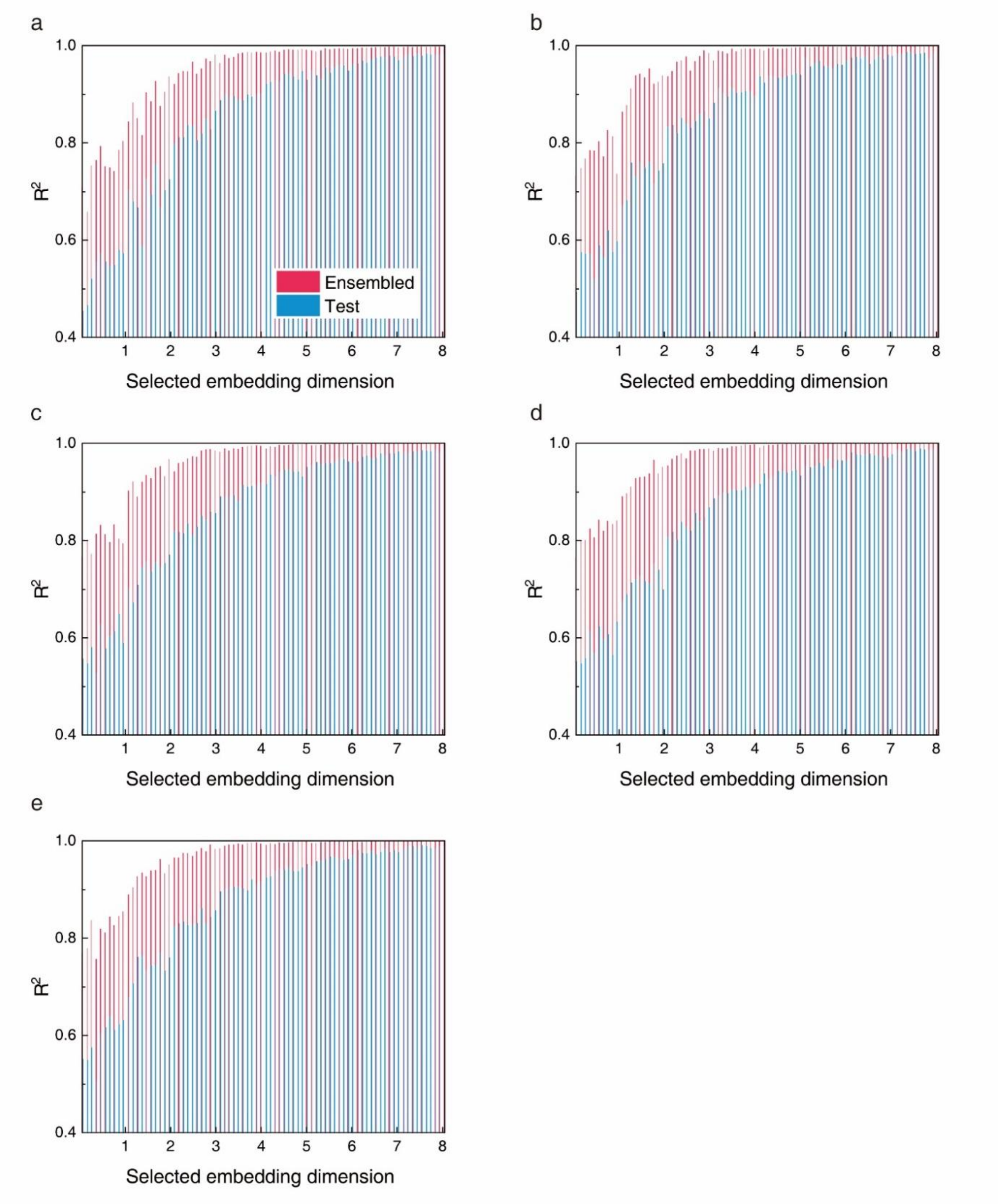

Supplementary Fig. 1. Comparison of embedding technique performance across cross-validation folds. (**a**) 3-folds, (**b**) 5-folds, (**c**) 7-folds, (**d**) 10-folds, and (**e**) 15-folds cross-validation. Ten distinct combinations of auto-encoder node counts were systematically tested For each embedding dimension ([8,8,8], [12,8,8], [12,12,12], [16,12,12], [16,16,16], [24,16,16], [24,24,24], [32,24,24], [32,32,32], and [36,36,36]), progressing from the simplest to the most complex configurations. Source data are provided as a Source Data file.

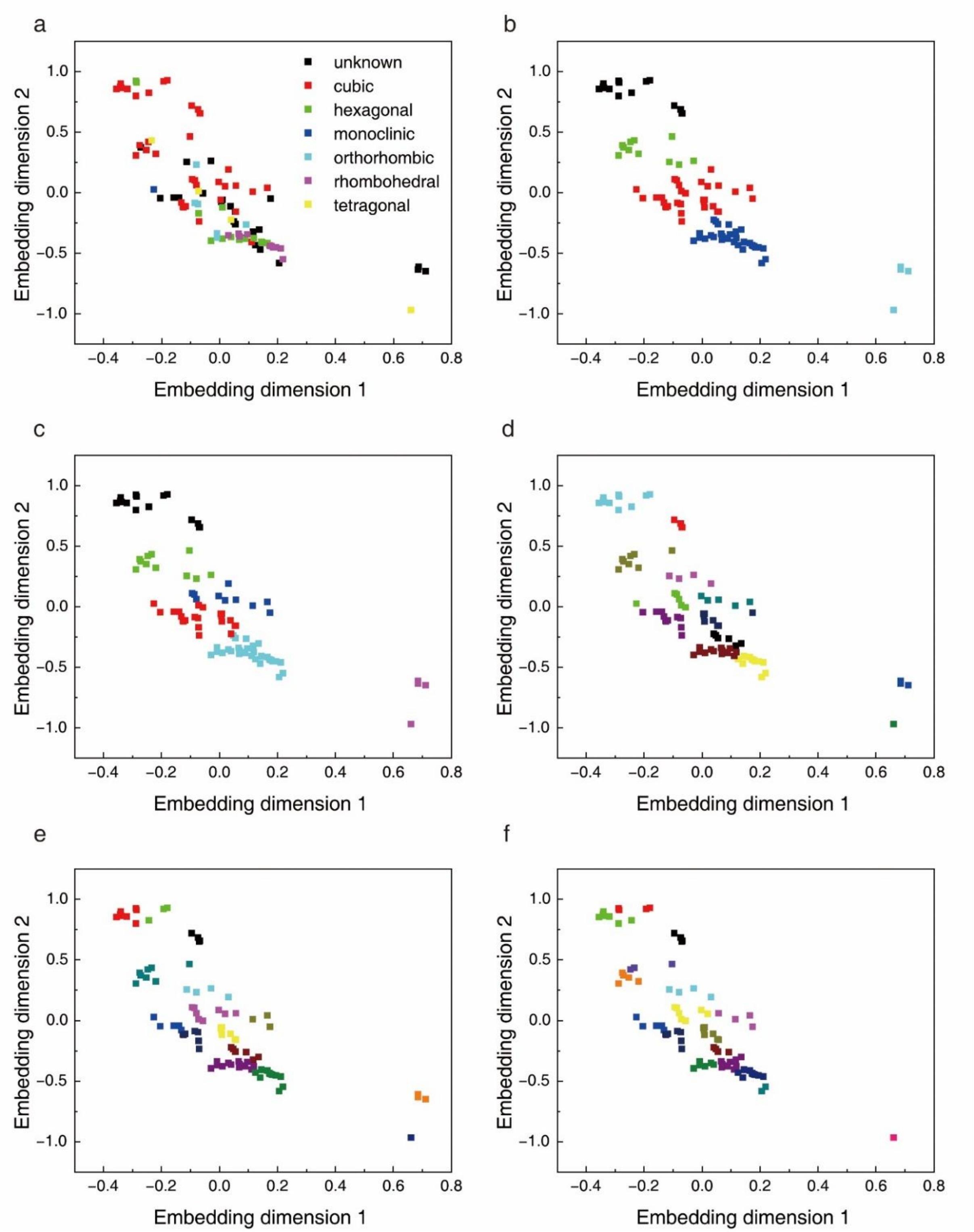

Supplementary Fig. 2. Data distribution visualization based on various clustering methods employed in the first round. (**a**) Phase-sorting 7 clusters, (**b**) K-Means 5 clusters, (**c**) K-Means 6 clusters, (**d**) K-Means 13 clusters, (**e**) K-Means 15 clusters and (**f**) K-Means 18 clusters. The color of each data point maps to its assigned cluster membership. Source data are provided as a Source Data file.

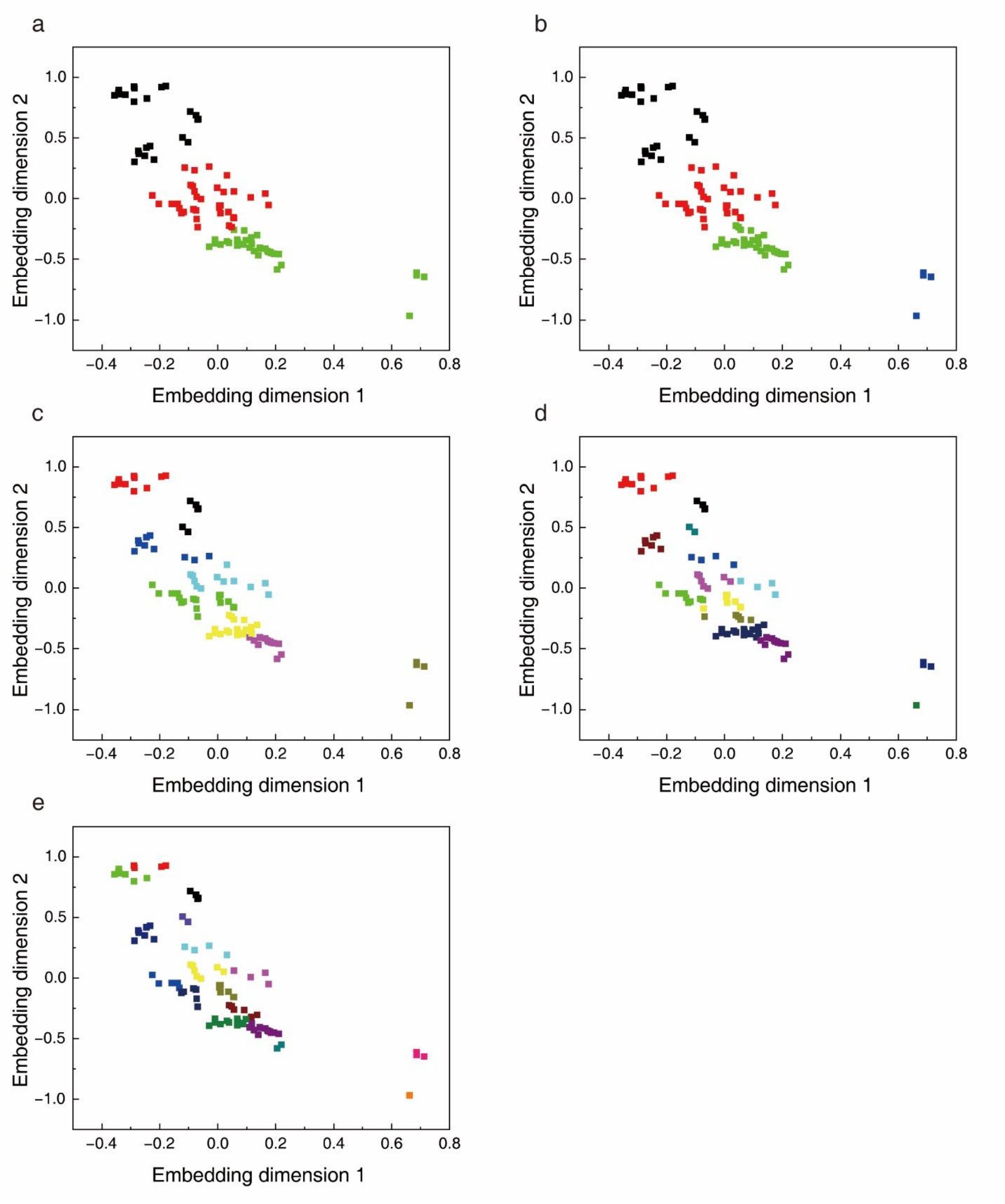

Supplementary Fig. 3. Data distribution visualization based on various clustering methods employed in the second round. (**a**) K-Means 3 clusters, (**b**) K-Means 4 clusters, (**c**) K-Means 8 clusters, (**d**) K-Means 14 clusters, and (**e**) K-Means 17 clusters. The color of each data point corresponds to its assigned cluster membership. Phase-sorting clustering is not presented as it does not change the cluster assignments. Source data are provided as a Source Data file.

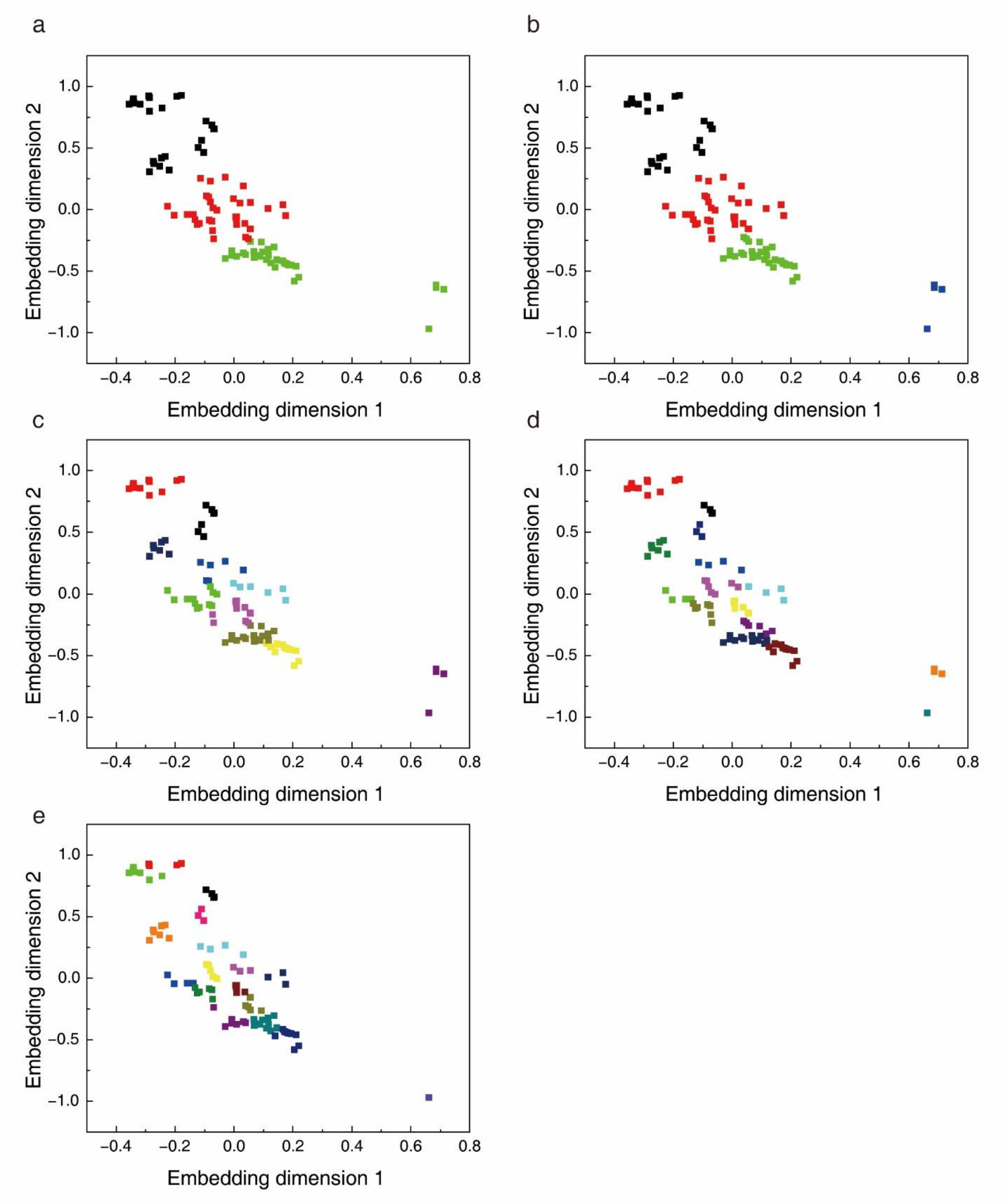

Supplementary Fig. 4. Data distribution visualization based on various clustering methods employed in the third round. (**a**) K-Means 3 clusters, (**b**) K-Means 4 clusters, (**c**) K-Means 10 clusters, (**d**) K-Means 15 clusters, and (**e**) K-Means 18 clusters. The color of each data point corresponds to its assigned cluster membership. Phase-sorting clustering is not presented as it does not change the cluster assignments. Source data are provided as a Source Data file.

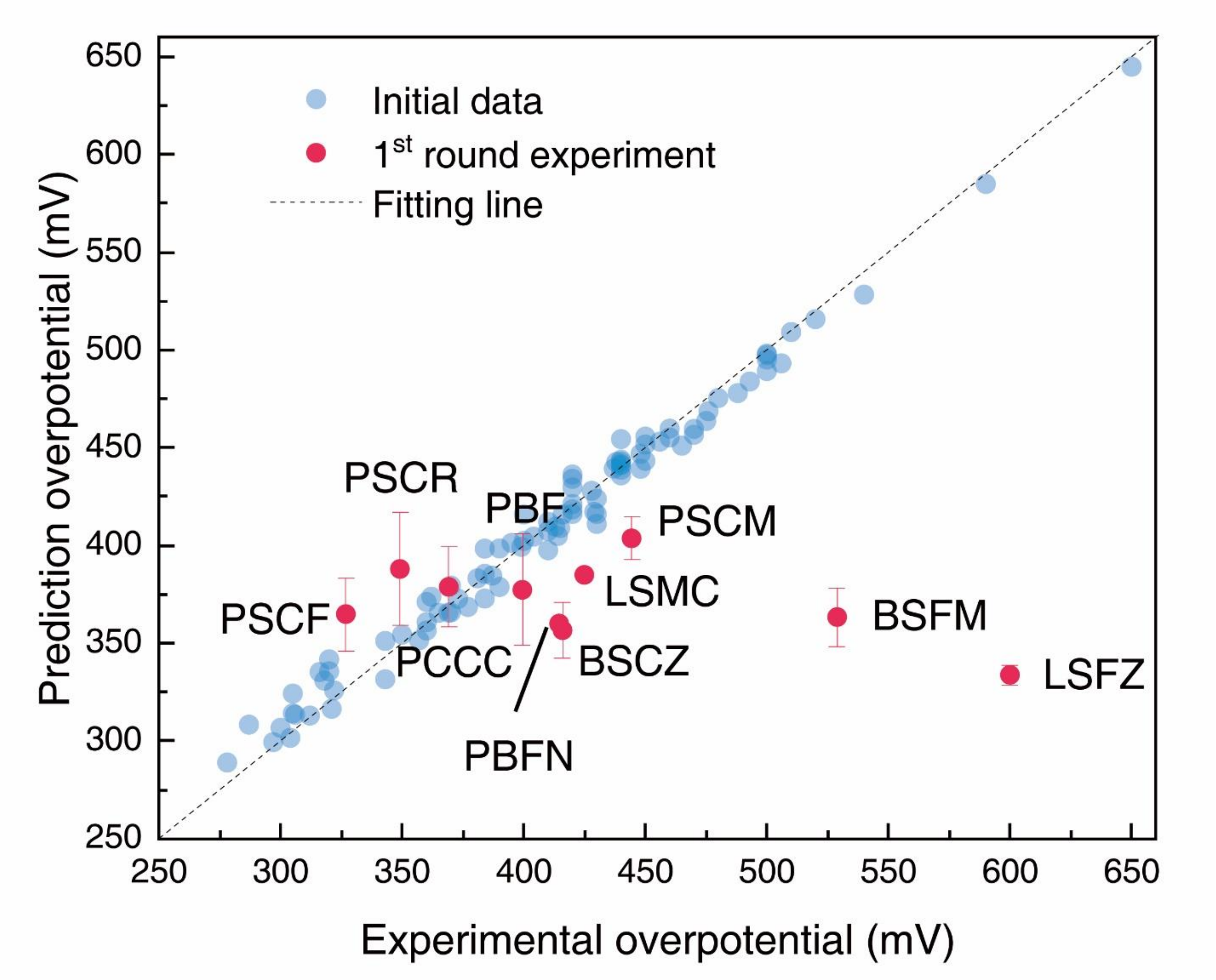

Supplementary Fig. 5. First round prediction of OER activities. All materials presented were both predicted and experimentally verified to be in a pure phase. $La_{0.7}Sr_{0.3}Fe_{0.5}Zr_{0.5}O_3$ (LSFZ) was assigned a true overpotential value of 600 mV due to the absence of a detected scanning signal (same below). The error bar represents the standard deviation of the prediction. Source data are provided as a Source Data file.

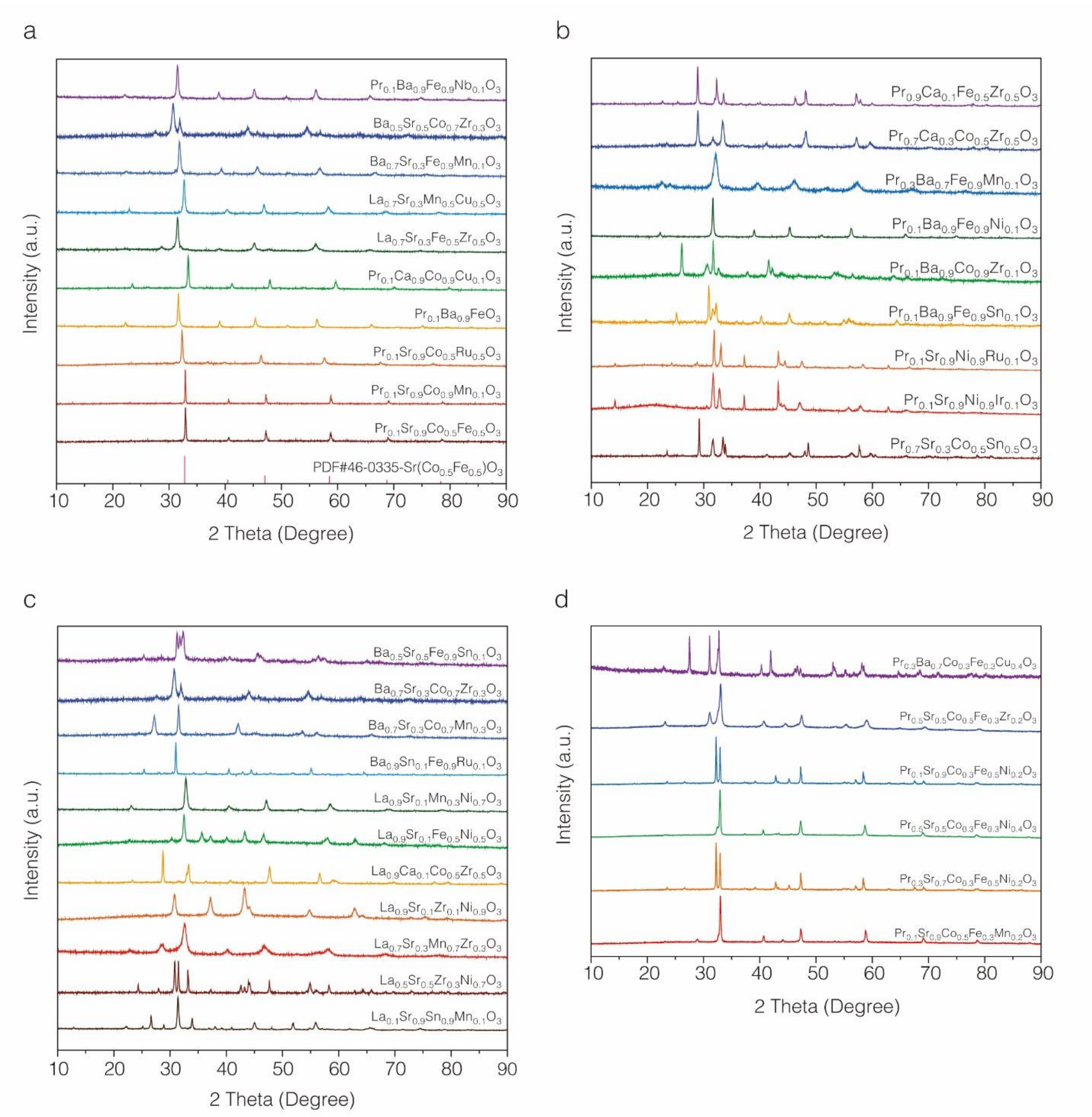

Supplementary Fig. 6. X-ray diffraction patterns of as-predicted perovskite compositions. (**a** to **c**) The XRD patterns of 36 perovskite-based electrocatalysts based on machine learning predictions. The materials shown in Fig. S6a exhibit a predominant perovskite phase according to the first round of prediction. The materials in Supplementary Fig. 6, b and c contain perovskite phases, but also significant impurities. (**d**) The materials shown in Supplementary Fig. 6d exhibit a predominant perovskite phase according to the second round of prediction. Source data are provided as a Source Data file.

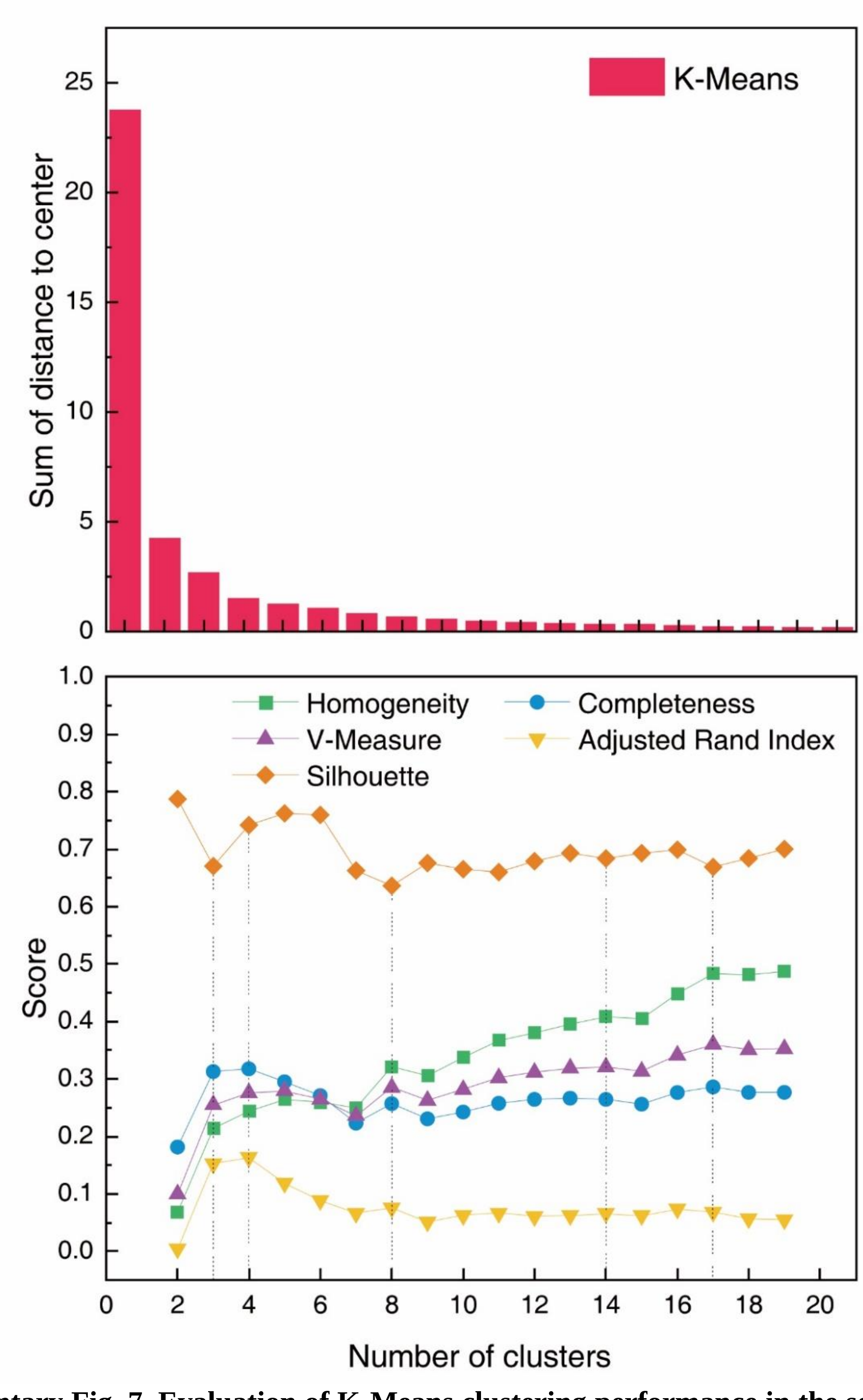

Supplementary Fig. 7. Evaluation of K-Means clustering performance in the second round. Multiple evaluation metrics are implemented, including the sum of distances to the cluster center, Adjusted Rand Index (ARI), V-Measure, Completeness, Homogeneity, and Silhouette Score. Optimal cluster sizes (3, 4, 8, 14, 17) are highlighted (by black dashed line) for their potential high performance. Source data are provided as a Source Data file.

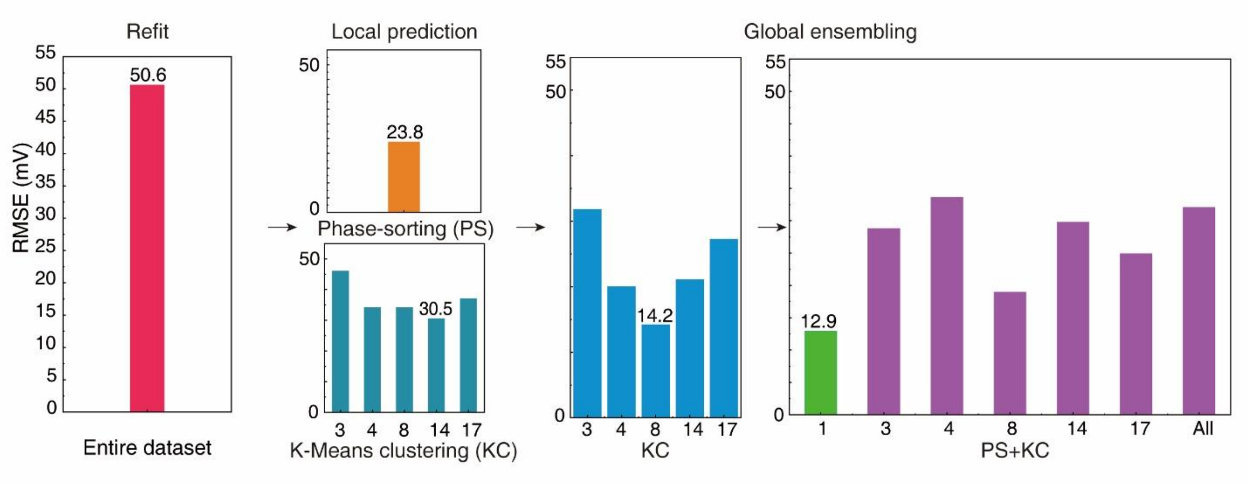

Supplementary Fig. 8. Prediction accuracy comparison for OER activities in the second round. Four different approaches are evaluated: direct refit model, local prediction model, globally ensembled model based on single clustering methods, and global model integrating multiple clustering methods. The best RMSE value (12.89 mV) was achieved by ensembling phase-sorting and non-clustering (1 cluster) together. Source data are provided as a Source Data file.

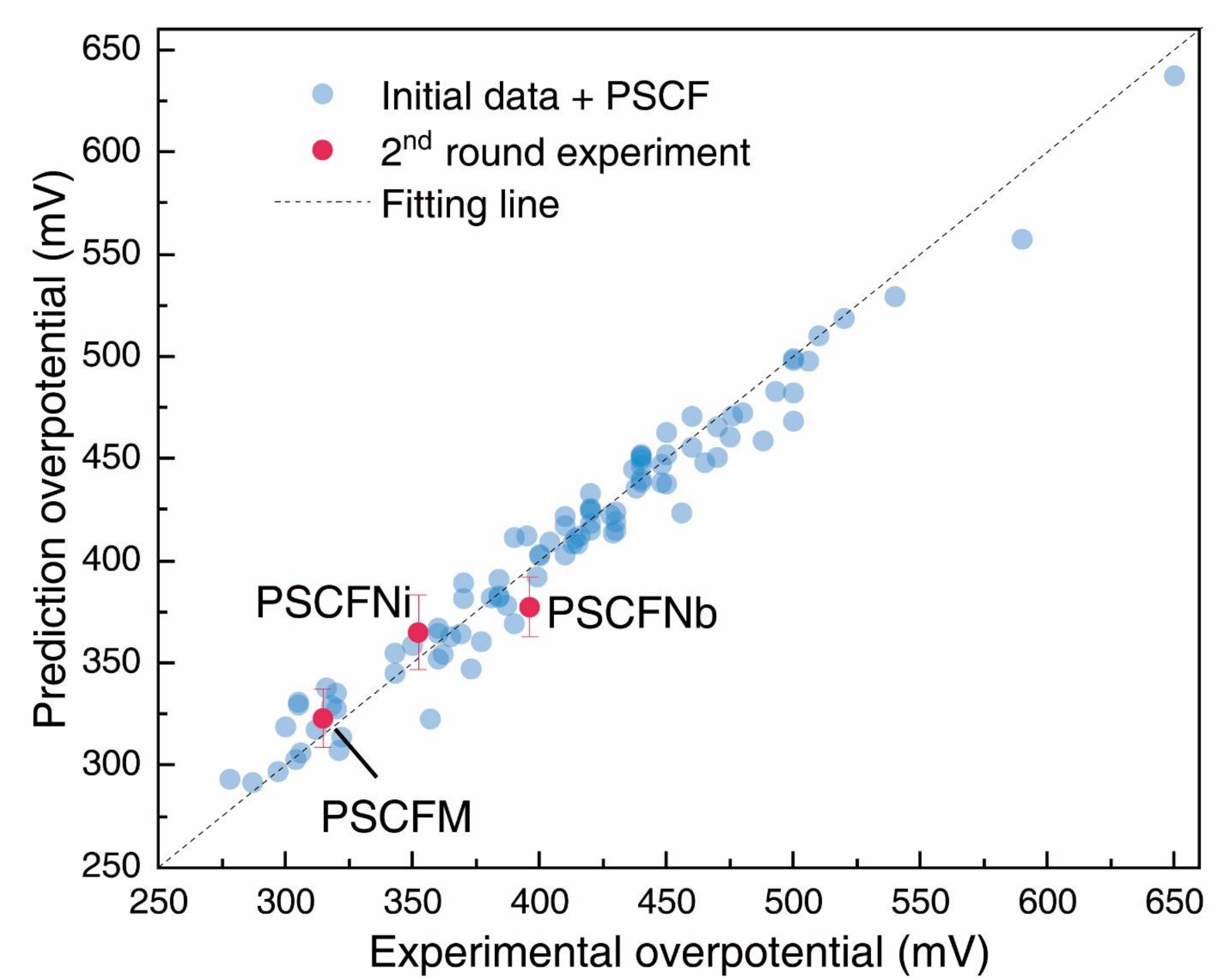

Supplementary Fig. 9. Second-round prediction of OER activities. All materials presented were both predicted and experimentally verified to be in a pure phase (including PSCF). The error bar represents the standard deviation of the prediction. Source data are provided as a Source Data file.

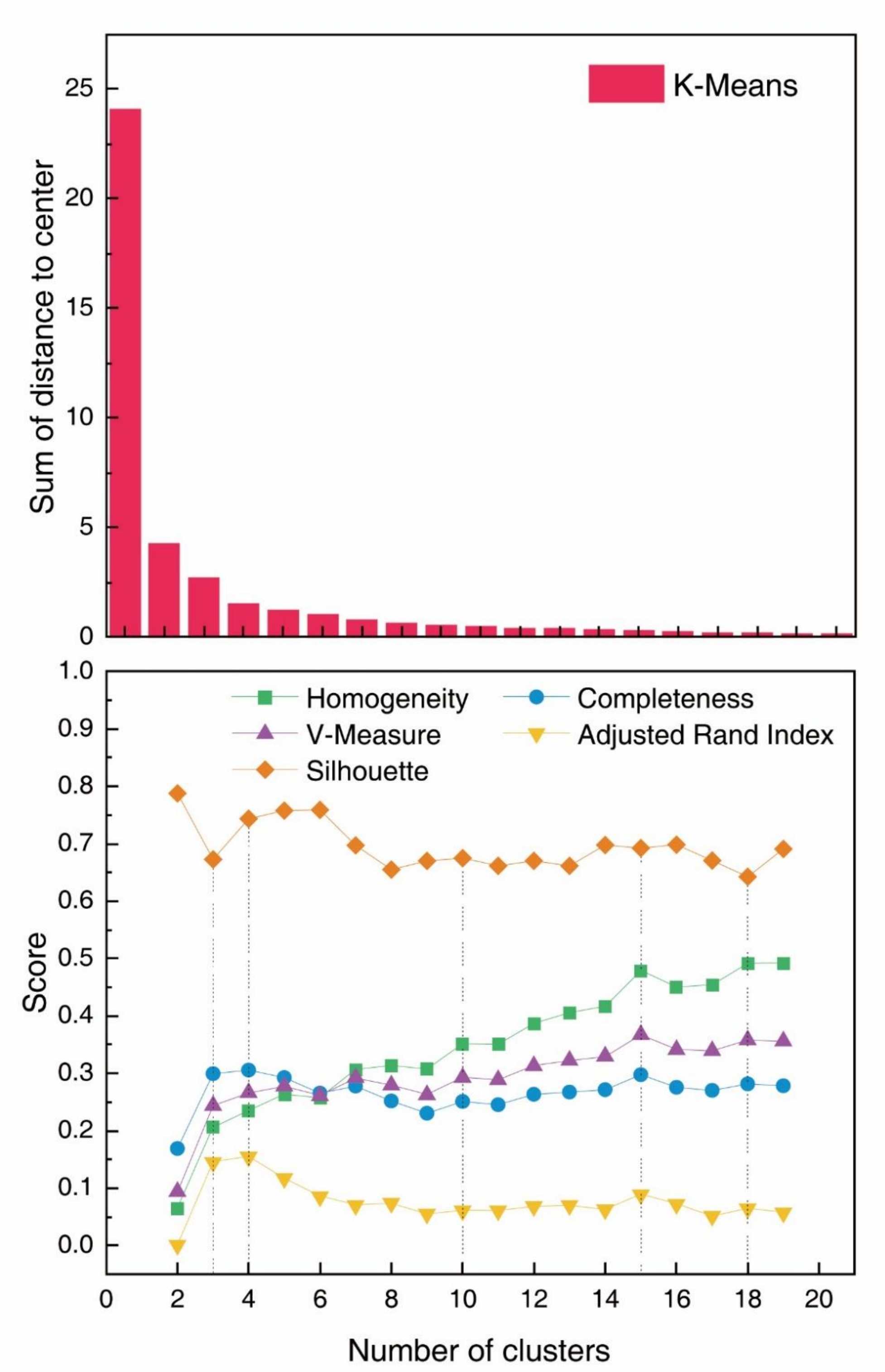

Supplementary Fig. 10. Evaluation of K-Means clustering performance in the third round. Multiple evaluation metrics are implemented, including the sum of distances to the center, Adjusted Rand Index (ARI), V-Measure, Completeness, Homogeneity, and Silhouette Score. Optimal cluster sizes (3, 4, 10, 15, 18) are highlighted (by black dashed line) for their potential high-performance. Source data are provided as a Source Data file.

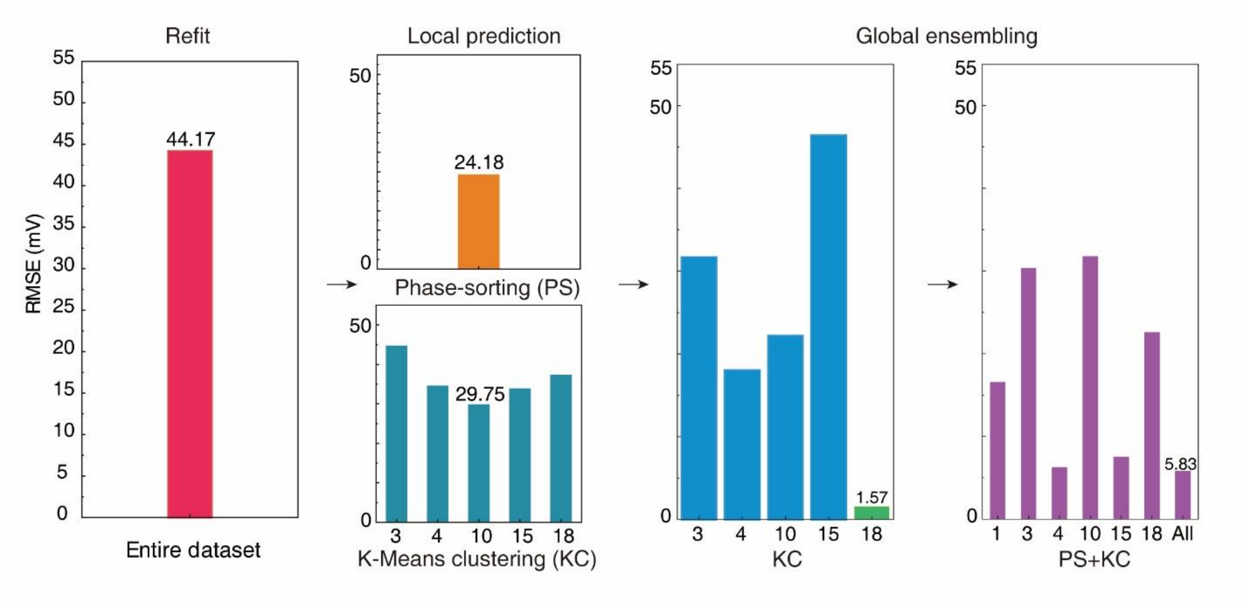

Supplementary Fig. 11. Prediction accuracy comparison for OER activities in the third round. Four different approaches are evaluated: direct refit models, local prediction models, globally ensembled models based on single clustering methods, and global models integrating multiple clustering methods. The best RMSE values (12.89 mV) were achieved by ensembling K-Means 18 clusters. Source data are provided as a Source Data file.

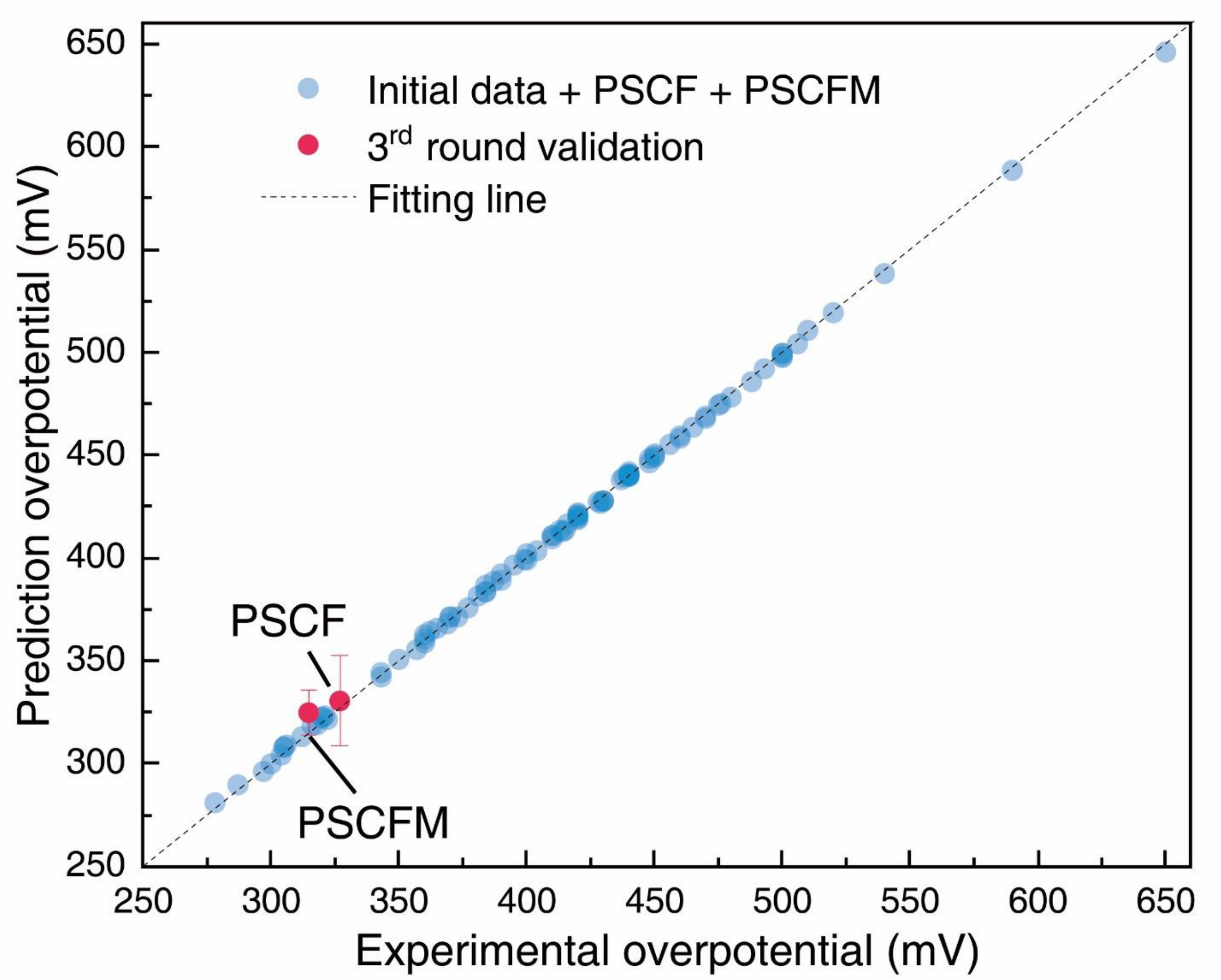

Supplementary Fig. 12. Third-round prediction of PSCF and PSCFM for active learning validation. The error bar represents the standard deviation of the prediction. Source data are provided as a Source Data file.

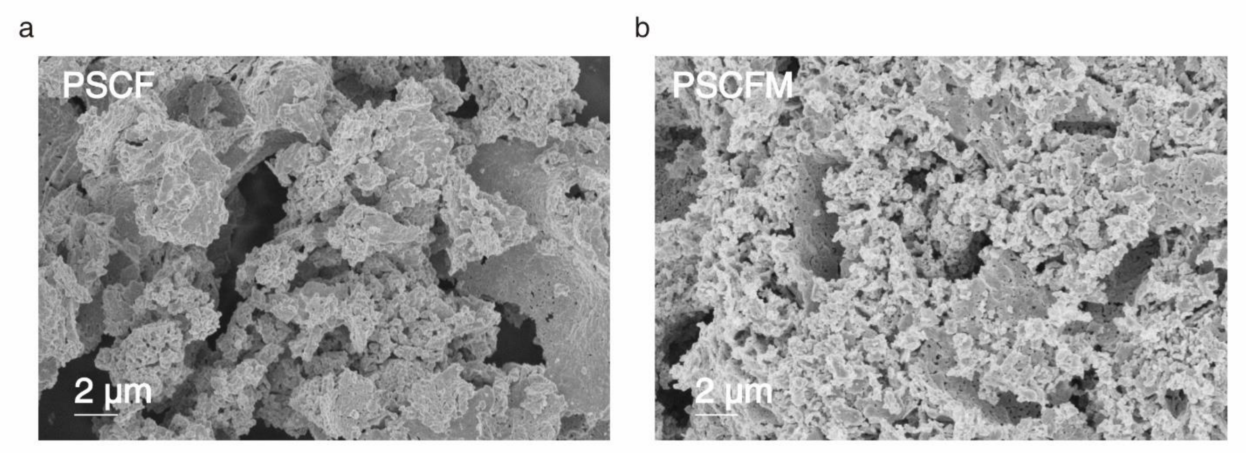

Supplementary Fig. 13. SEM images of (a) PSCF and (b) PSCFM.

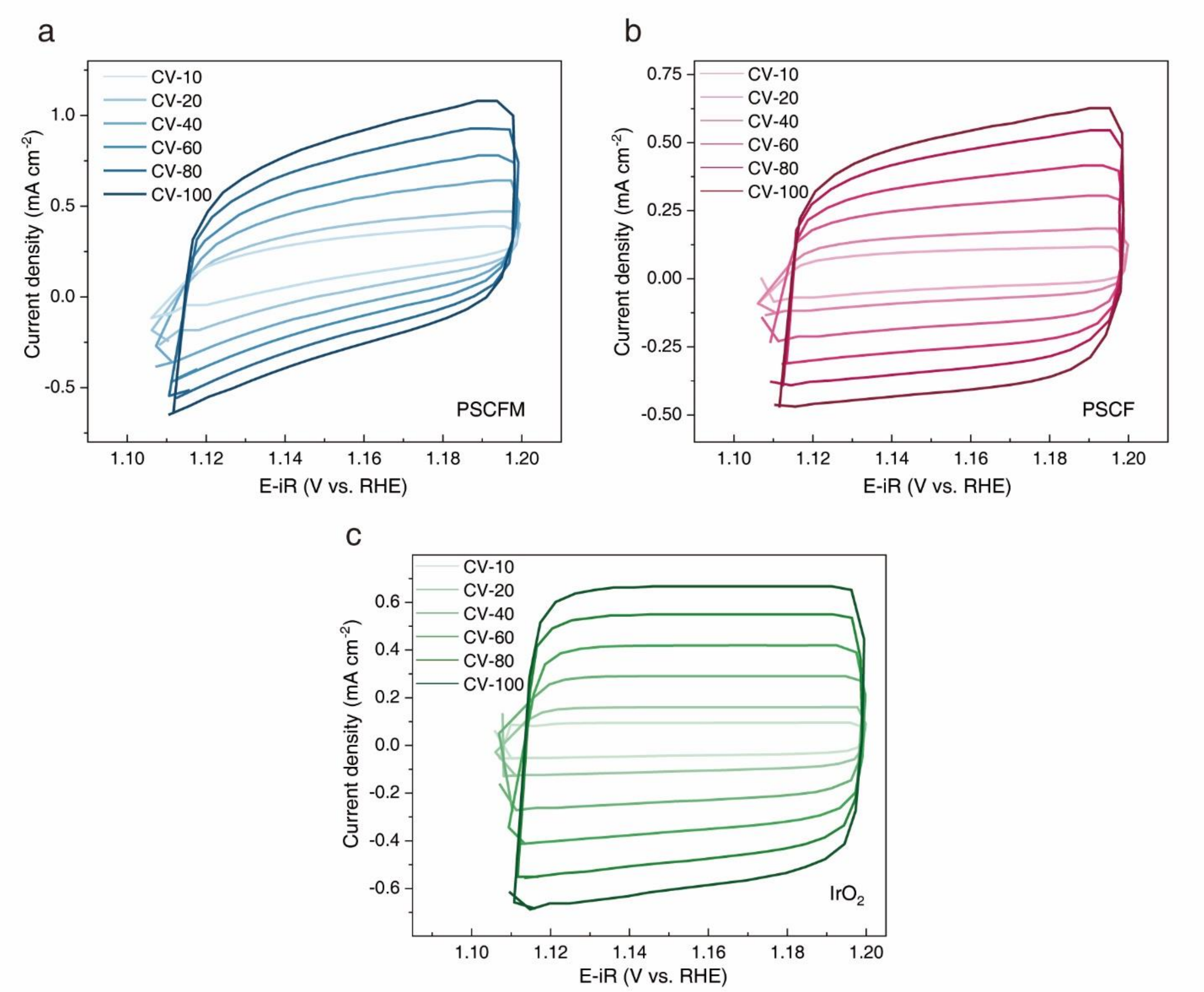

Supplementary Fig. 14. Cyclic voltammetry scans at different scan rates for (a) PSCFM, (b) PSCF and (c) IrO_2. The potential window is from 1.105 V to 1.205 V. The potentials were iR corrected and the R values for IrO_2, PSCF and PSCFM measurements were 5.98 ± 0.07, 6.0 ±0.04 and 6.0 ±0.06 Ω, respectively. Source data are provided as a Source Data file.

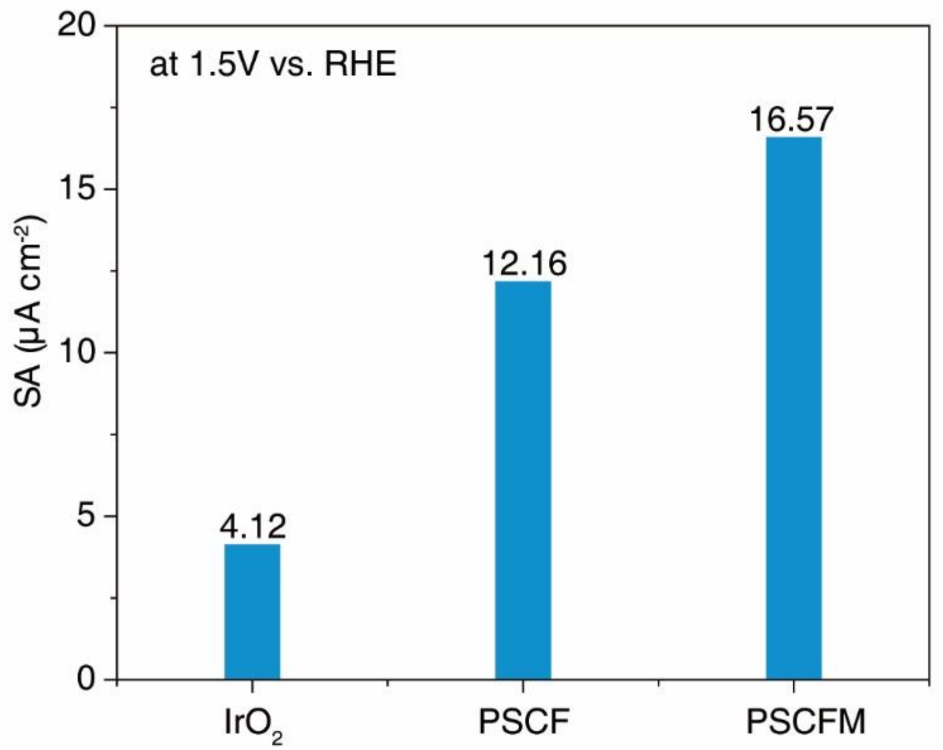

Supplementary Fig. 15. ESCA normalized activity of electrocatalysts at 1.5 V vs. RHE. Source data are provided as a Source Data file.

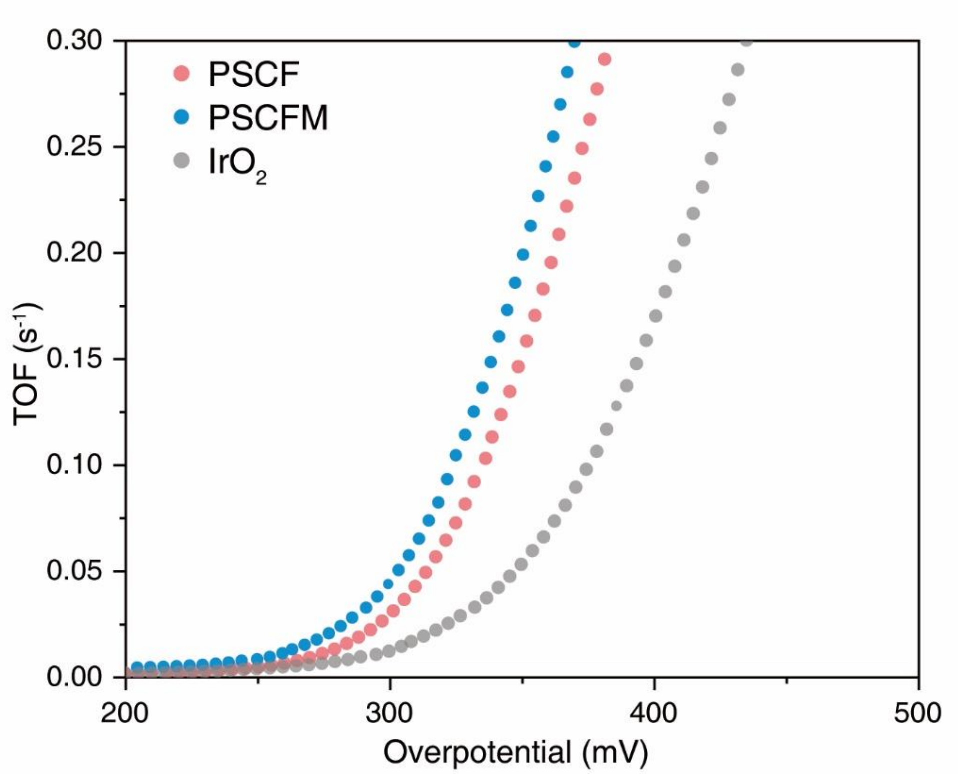

Supplementary Fig. 16. TOF plot of different electrocatalysts. Source data are provided as a Source Data file.

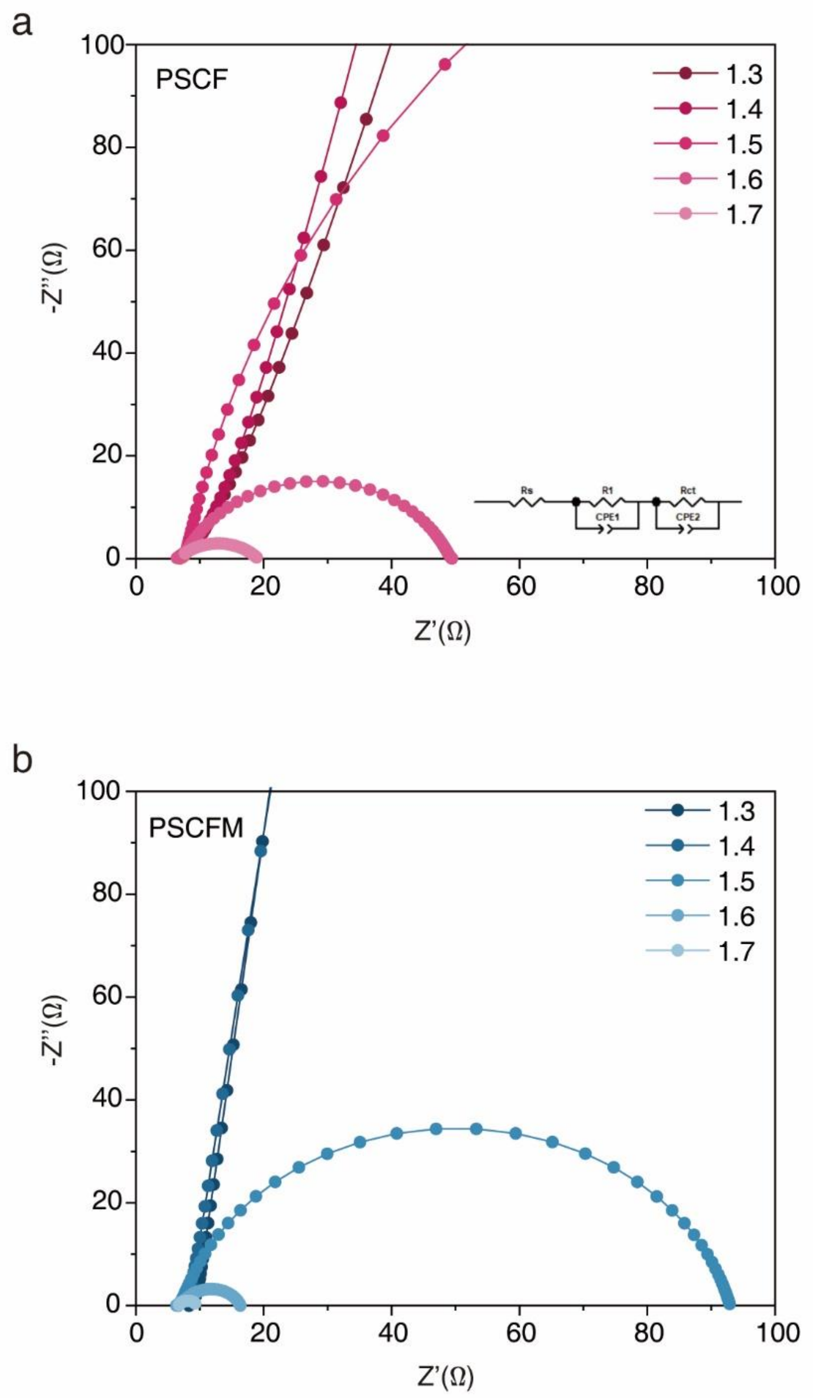

Supplementary Fig. 17. In-situ EIS plots of (a) PSCF and (b) PSCFM (1.3 V to 1.7 V vs. RHE). The inset section represents an analogue circuit diagram. Source data are provided as a Source Data file.

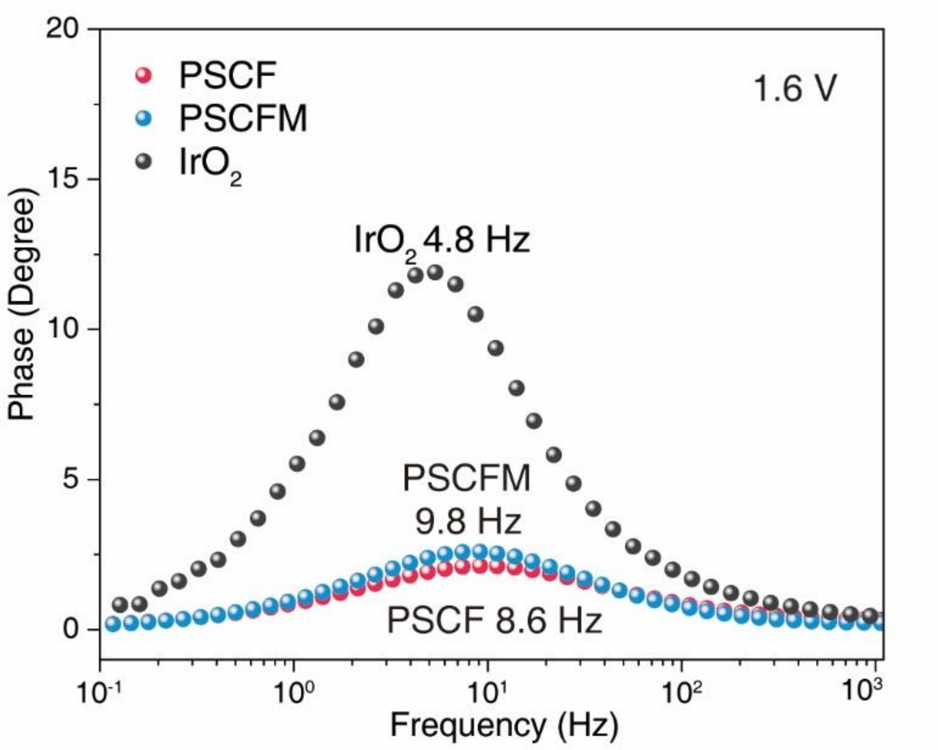

Supplementary Fig. 18. Bode plots of electrocatalysts. Source data are provided as a Source Data file.

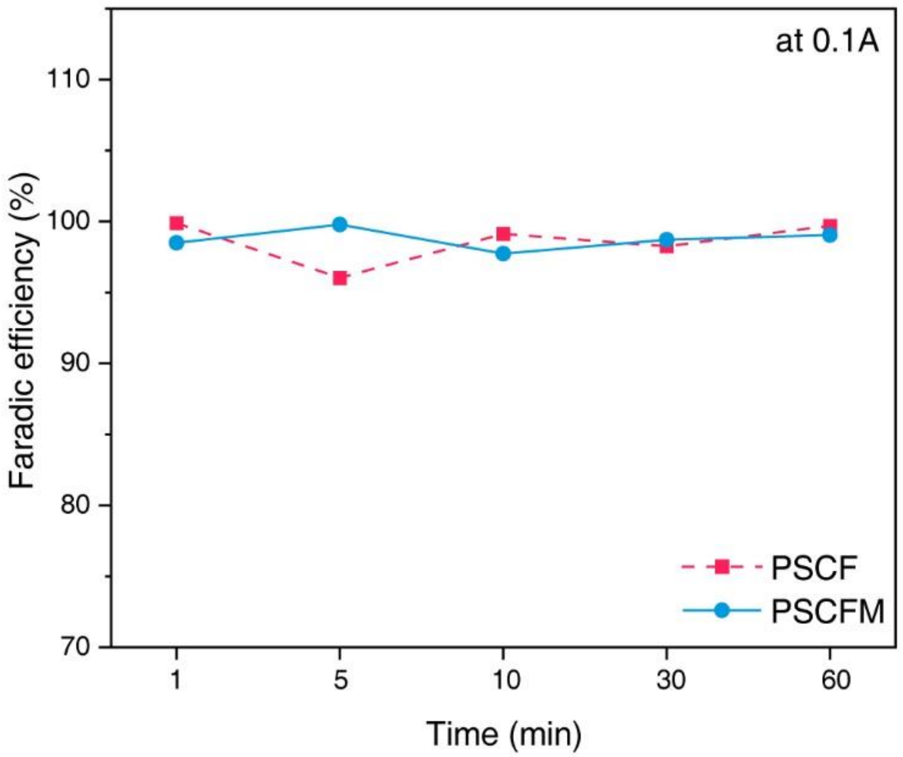

Supplementary Fig. 19. Faraday efficiency of PSCF and PSCFM at different electrolysis times (1 min, 5 min, 10 min, 30 min and 1 h). Source data are provided as a Source Data file.

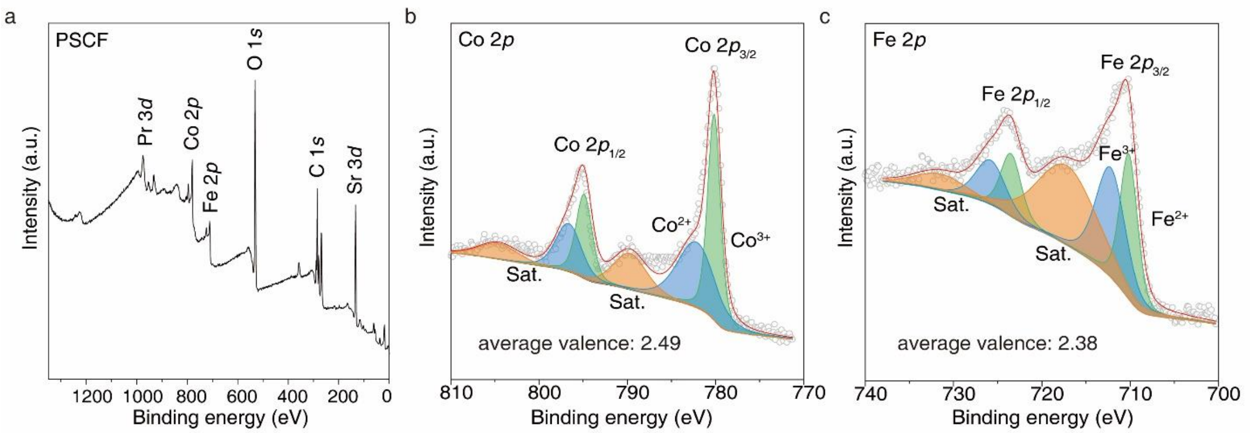

Supplementary Fig. 20. XPS spectra of PSCF. (**a**) The all survey of XPS of PSCF. (**b**) The Co 2*p* XPS spectrum. (**c**) The Fe 2*p* XPS spectrum. Source data are provided as a Source Data file.

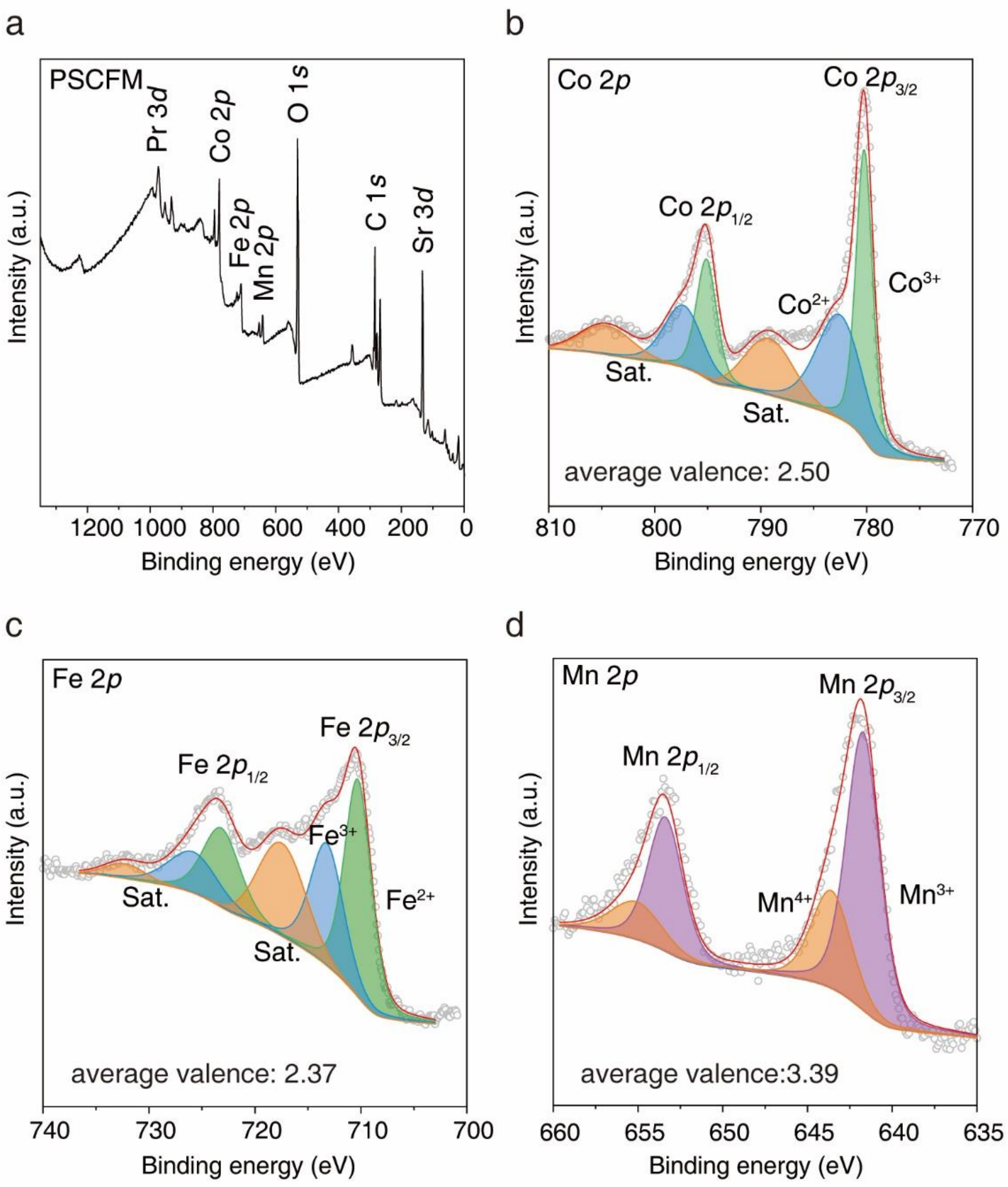

Supplementary Fig. 21. XPS spectra of PSCFM. (**a**) The all survey of XPS of PSCFM. (**b**) The Co 2*p* XPS spectrum. (**c**) The Fe 2*p* XPS spectrum. (**d**) The Mn 2*p* XPS spectrum. Source data are provided as a Source Data file.

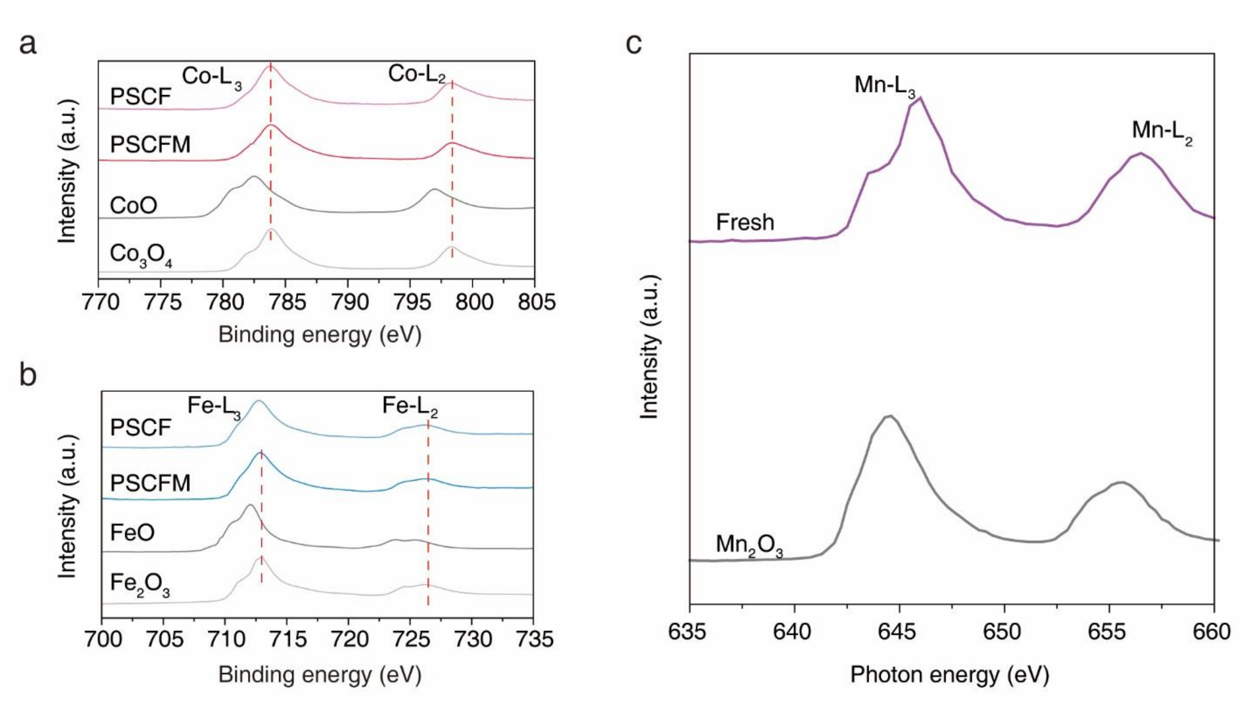

Supplementary Fig. 22. Soft XAS spectra of electrocatalysts. L-edge XAS of (a) Co, (b) Fe and (c) Mn in different electrocatalysts. Source data are provided as a Source Data file.

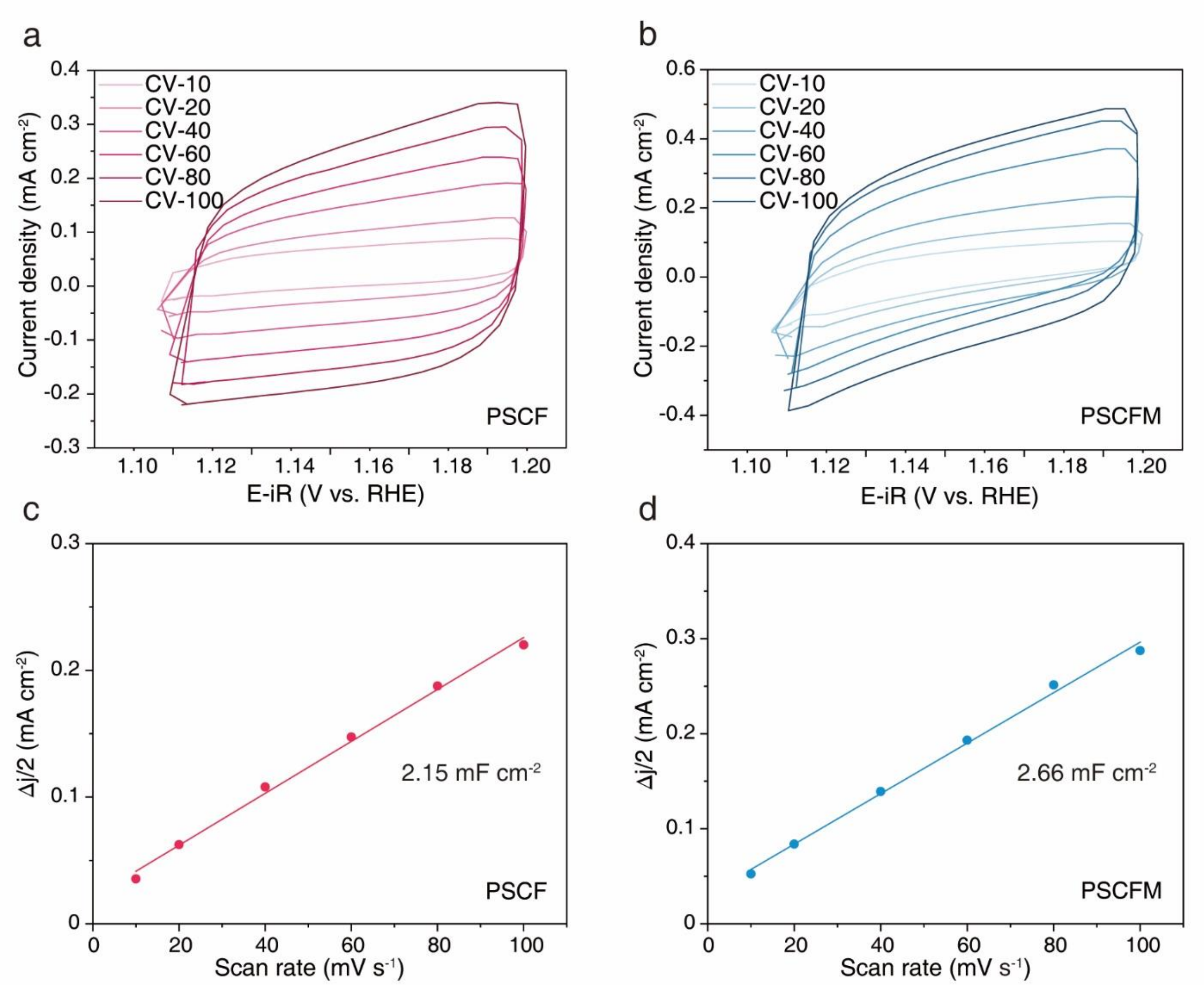

Supplementary Fig. 23. Double-layer capacitance (C_{dl}) determination for PSCF and PSCFM. The CV scans at different scan rates for (**a**) PSCF and (**b**) PSCFM in methanol containing KOH electrolyte. The potentials were iR corrected and the R values for PSCF and PSCFM measurements were 6.0 ±0.04 and 6.0 ±0.06 Ω, respectively. The Δj/2 vs. scan rate plot of (**c**) PSCF and (**d**) PSCFM electrodes. Source data are provided as a Source Data file.

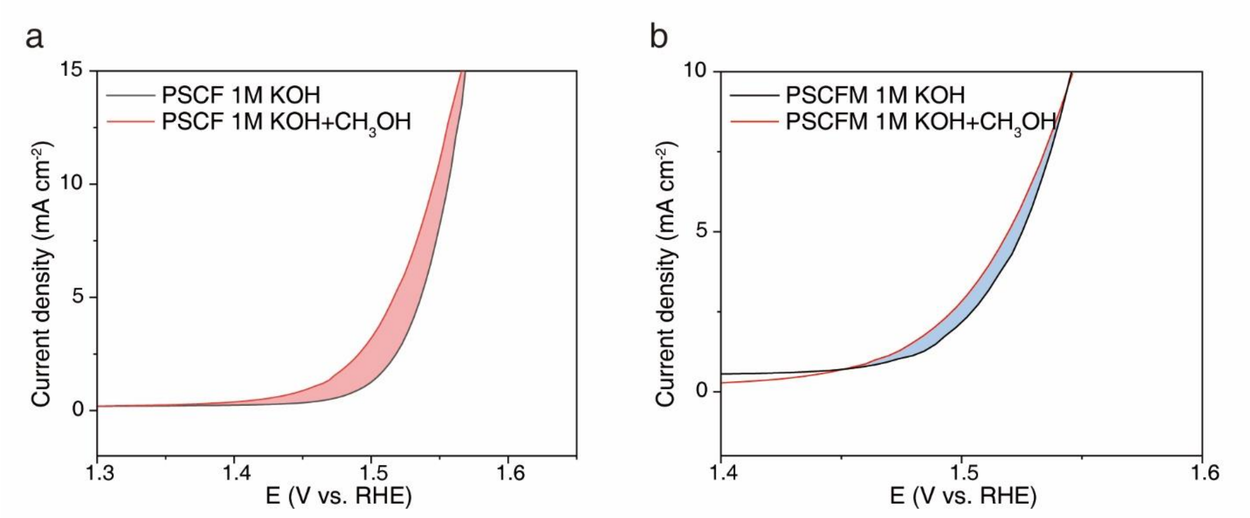

Supplementary Fig. 24. OER and MOR activities of PSCF and PSCFM. Linear sweep voltammetry curves of PSCF and PSCFM in 1.0 M KOH (**a**) with and (**b**) without methanol (0.602 mol L^{-1}) without iR-correction. Source data are provided as a Source Data file.

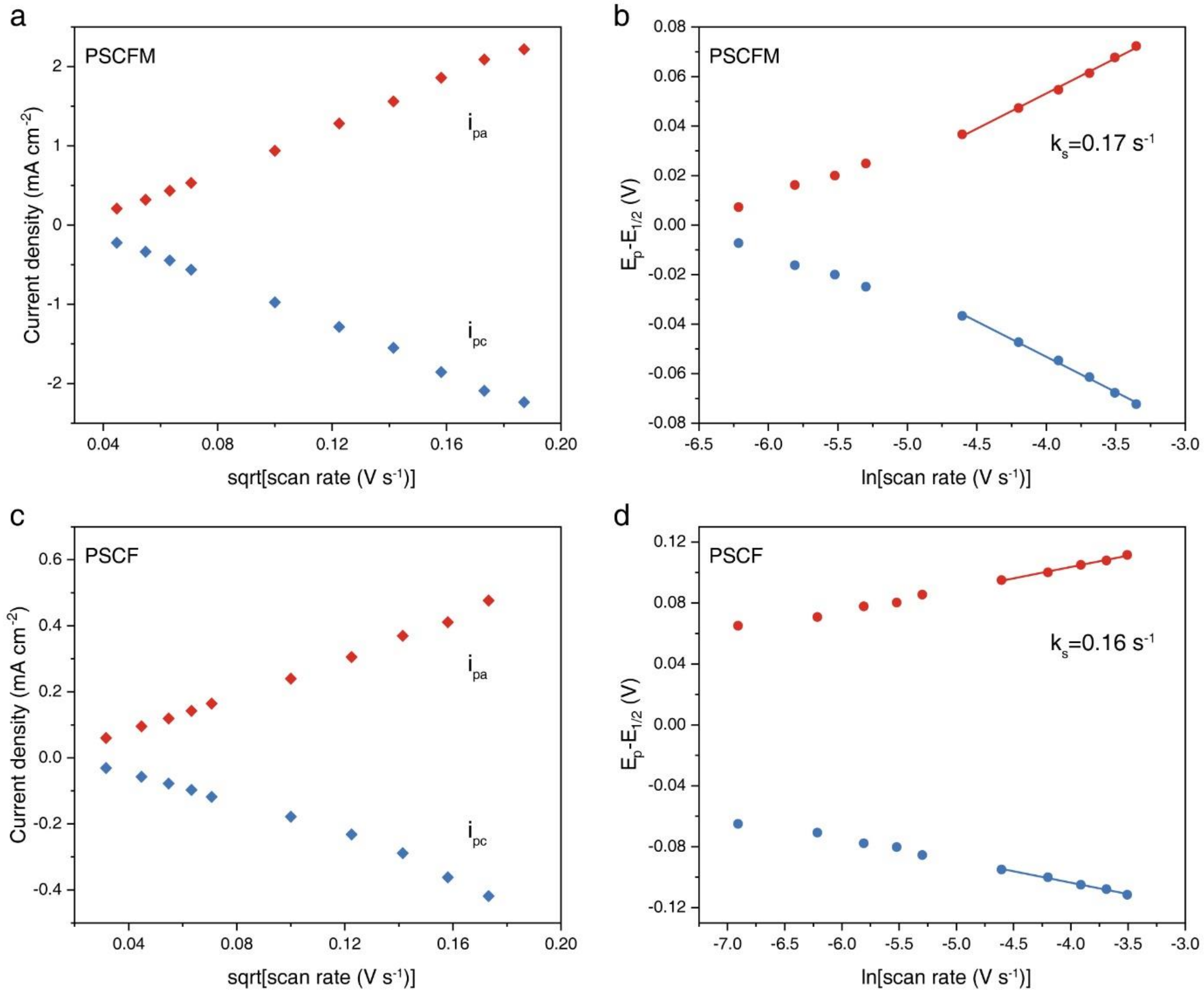

Supplementary Fig. 25. K_s of PSCF and PSCFM obtained from Laviron analysis. Plots of the redox peak current densities versus the square root of scan rates for (**a**) PSCFM and (**c**) PSCF. Laviron analyses of (**b**) PSCFM and (**d**) PSCF. Source data are provided as a Source Data file.

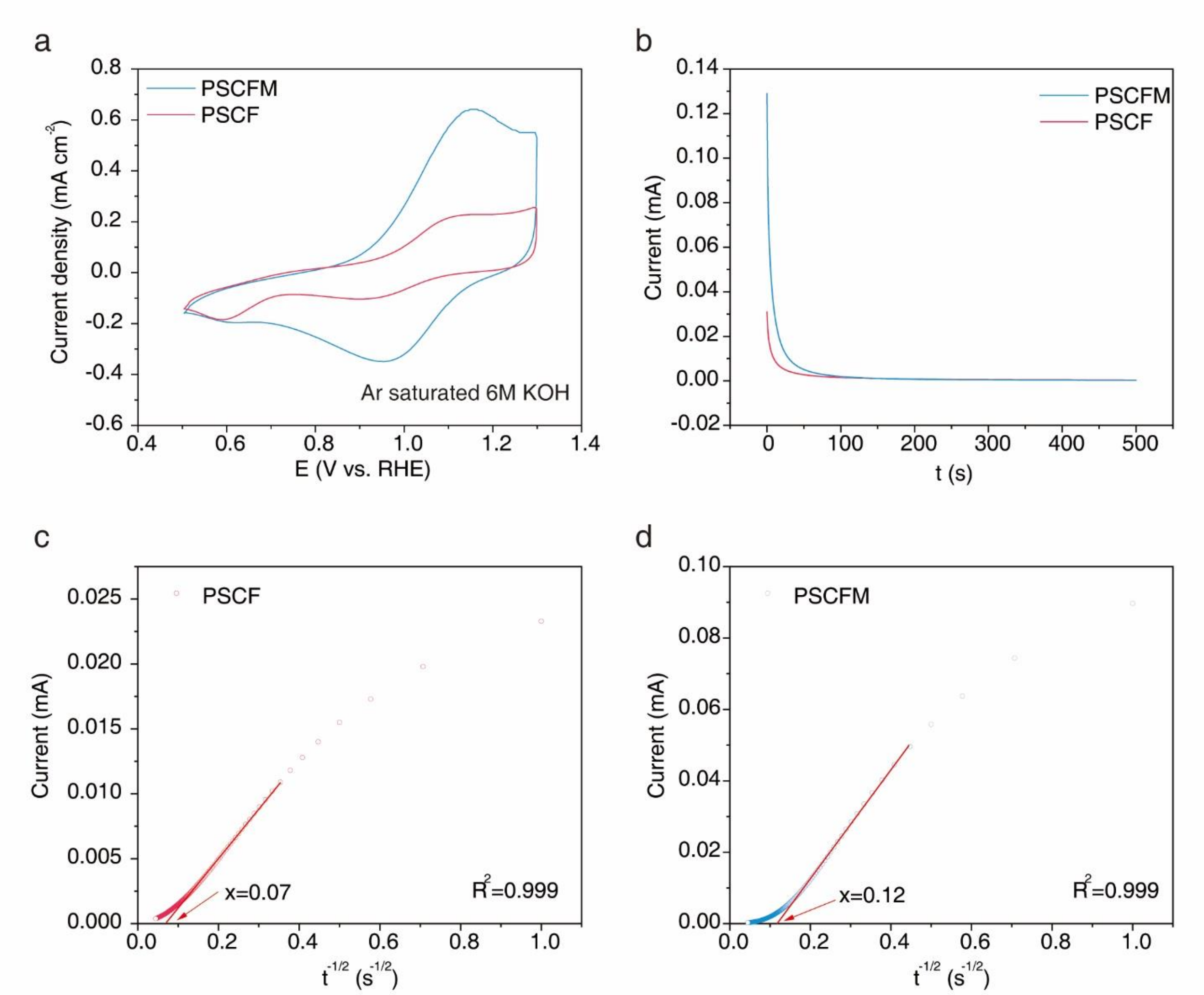

Supplementary Fig. 26. Characterization of oxygen ion diffusion coefficients for PSCF and PSCFM. (**a**) CV curves of PSCF and PSCFM in Ar-saturated 6 M KOH, where redox peaks indicate the electrochemical oxygen intercalation/deintercalation without iR-correction. (**b**) The corresponding current vs. time plot. (**c to d**) chronoamperometry data (i vs. $t^{-1/2}$) used for the calculation of oxygen ion diffusion coefficients. Source data are provided as a Source Data file.

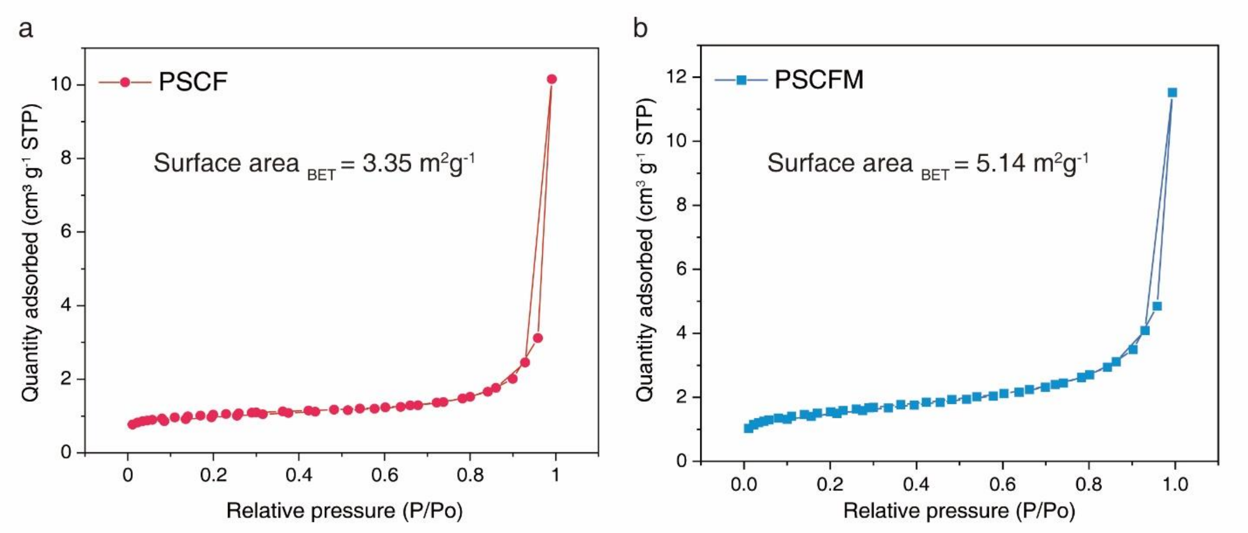

Supplementary Fig. 27. BET surface area of PSCF and PSCFM. N_2 absorption-desorption curves of (**a**) PSCF and (**b**) PSCFM samples. Source data are provided as a Source Data file.

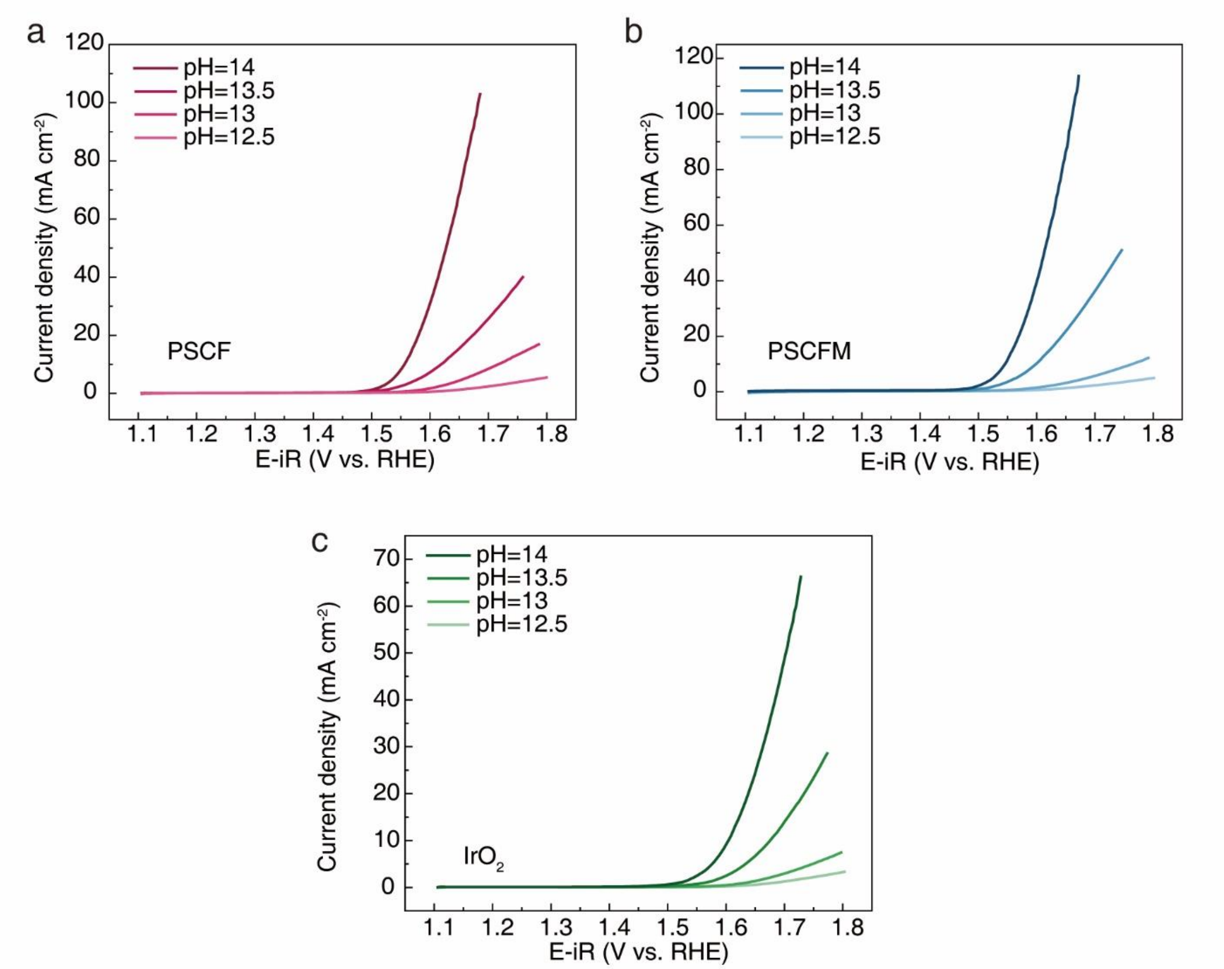

Supplementary Fig. 28. LSV curves in KOH electrolyte at different pH values of (a) PSCF and (b) PSCFM and (c) IrO_2. The potentials were iR corrected, and the R values for IrO_2, PSCF and PSCFM measurements were 5.98 ± 0.07, 6.0 ±0.04 and 6.0 ±0.06 Ω in KOH solution at pH 14.0 ± 0.03, respectively. The potentials were iR corrected, and the R values for IrO_2, PSCF and PSCFM measurements were 7.94 ± 0.04, 7.93 ±0.03 and 7.96 ±0.03 Ω in KOH solution at pH 13.5 ± 0.03, respectively. The potentials were iR corrected, and the R values for IrO_2, PSCF and PSCFM measurements were 11.6 ± 0.06, 11.7 ±0.08 and 11.6 ±0.08 Ω in KOH solution at pH 13.0 ± 0.03, respectively. The potentials were iR corrected, and the R values for IrO_2, PSCF and PSCFM measurements were 29.0 ± 0.1, 29.0 ±0.08 and 29.2 ±0.08 Ω in KOH solution at pH 12.5 ± 0.02, respectively. Source data are provided as a Source Data file.

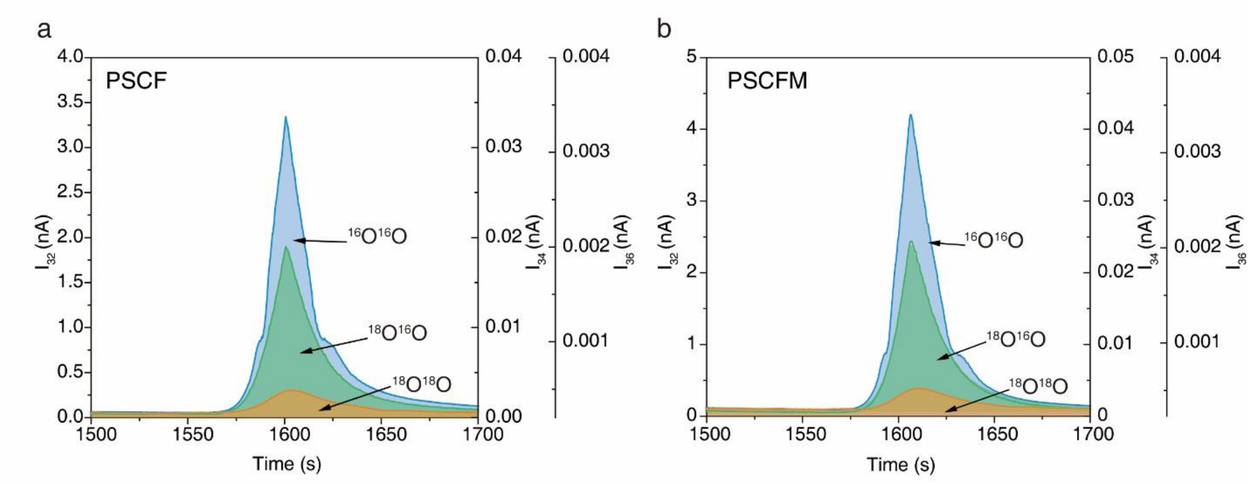

Supplementary Fig. 29. DEMS signals of $^{16}O_2$ (I_{32}), $^{16}O^{18}O$ (I_{34}), and $^{18}O_2$ (I_{36}) of (a) PSCF and (b) PSCFM. Source data are provided as a Source Data file.

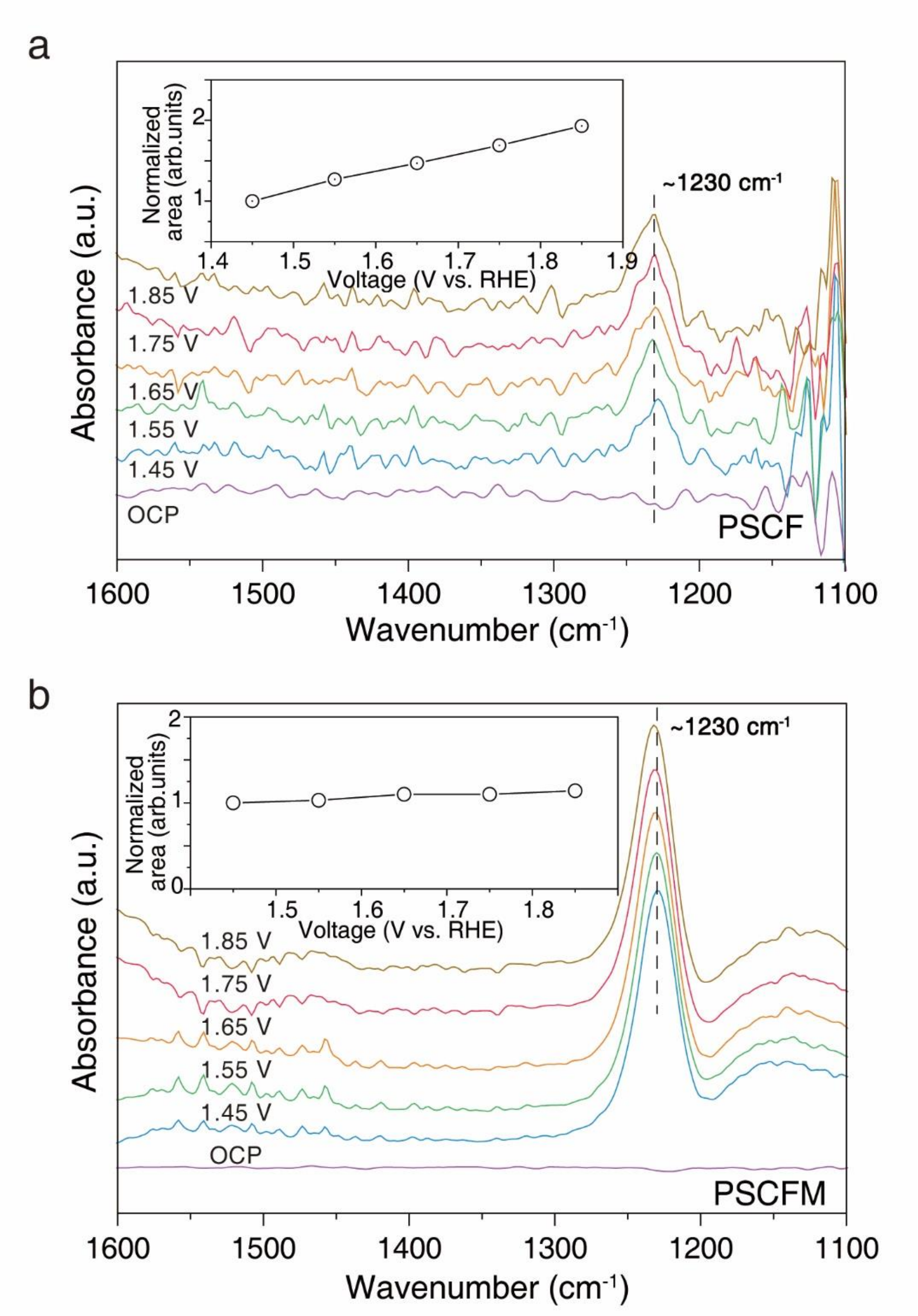

Supplementary Fig. 30. *In situ* ATR-IR spectra of (a) PSCF and (b) PSCFM under multi-potential steps. The insets summarize the normalized peak area (normalized to the area at 1.45 V) at 1230 cm^{-1} which corresponds to the *OOH intermediate. Obviously, the area of PSCF keeps increasing from 1.45 V to 1.85 V, while that of PSCFM does not show apparent fluctuation. Such huge difference indicates that PSCFM has significant larger amount of *OOH which quickly saturates at lower overpotential. Source data are provided as a Source Data file.

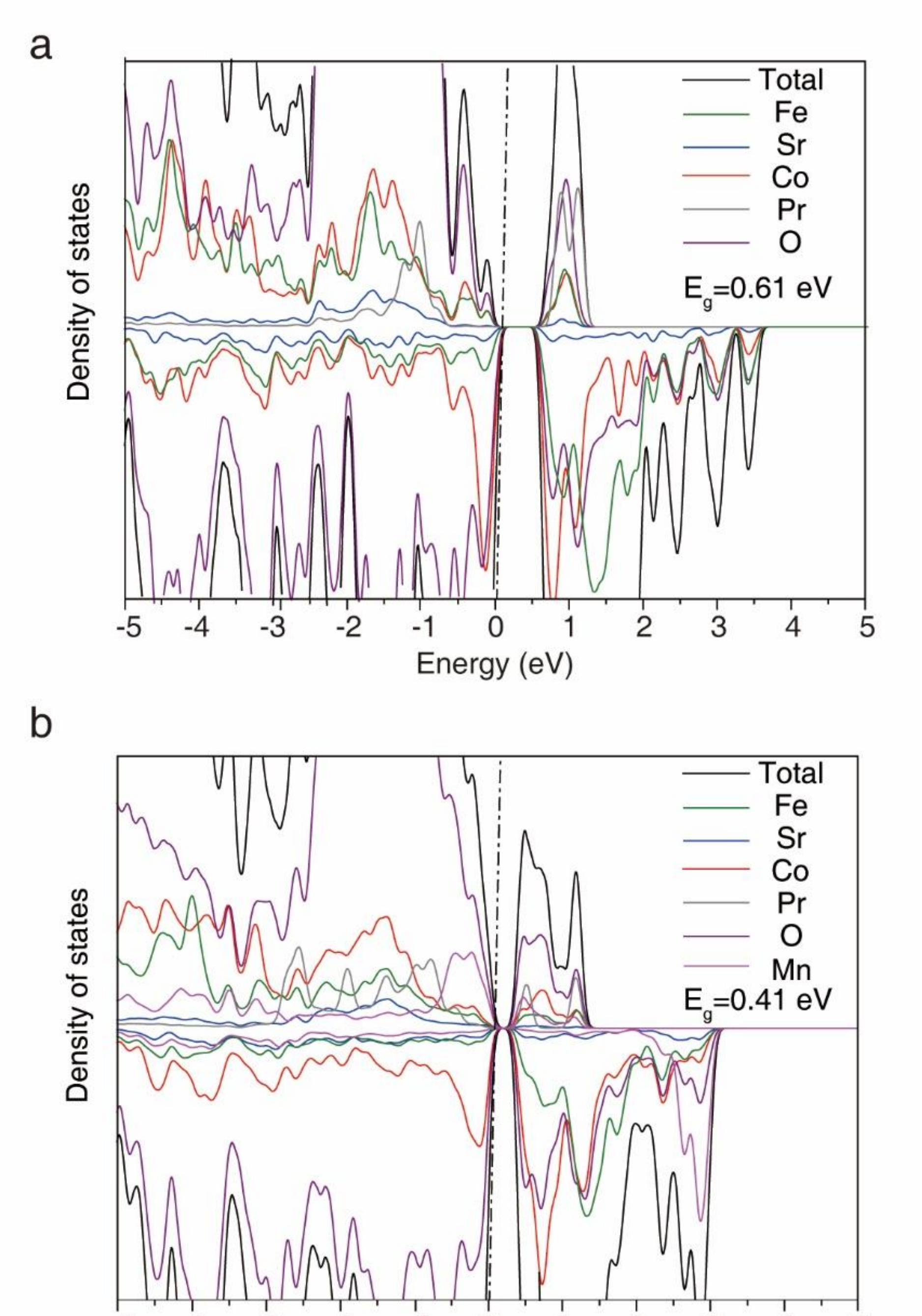

Supplementary Fig. 31. Projected density of states of (a) PSCF and (b) PSCFM. The calculated bandgap of PSCF and PSCFM is 0.61 eV and 0.41 eV, respectively. Source data are provided as a Source Data file.

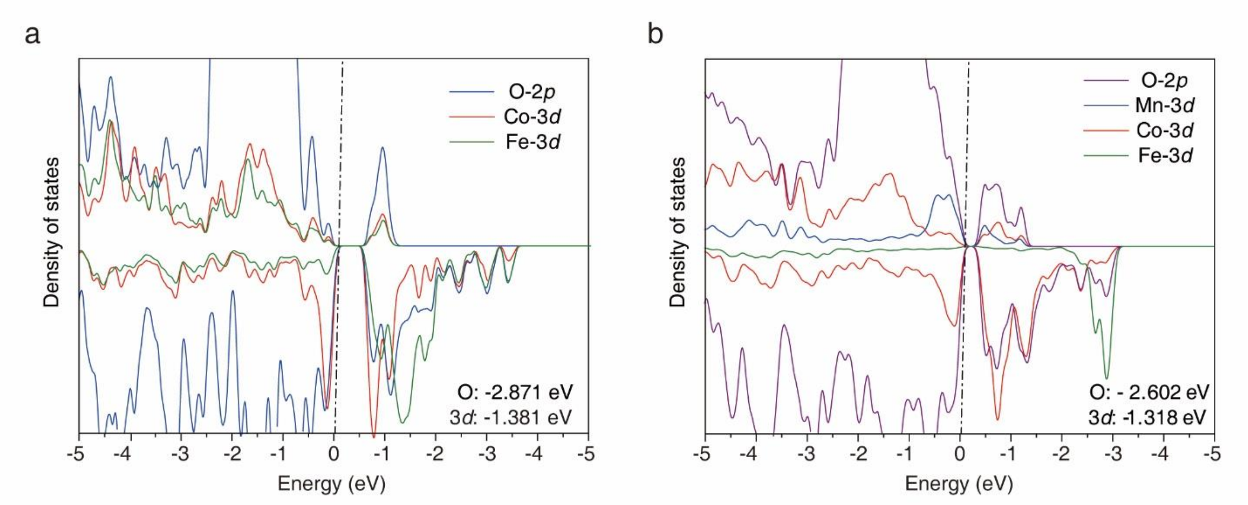

Supplementary Fig. 32. Projected density of states of O 2*p*, Co 3*d* and Fe 3*d* of (a) PSCF and (b) PSCFM. Source data are provided as a Source Data file.

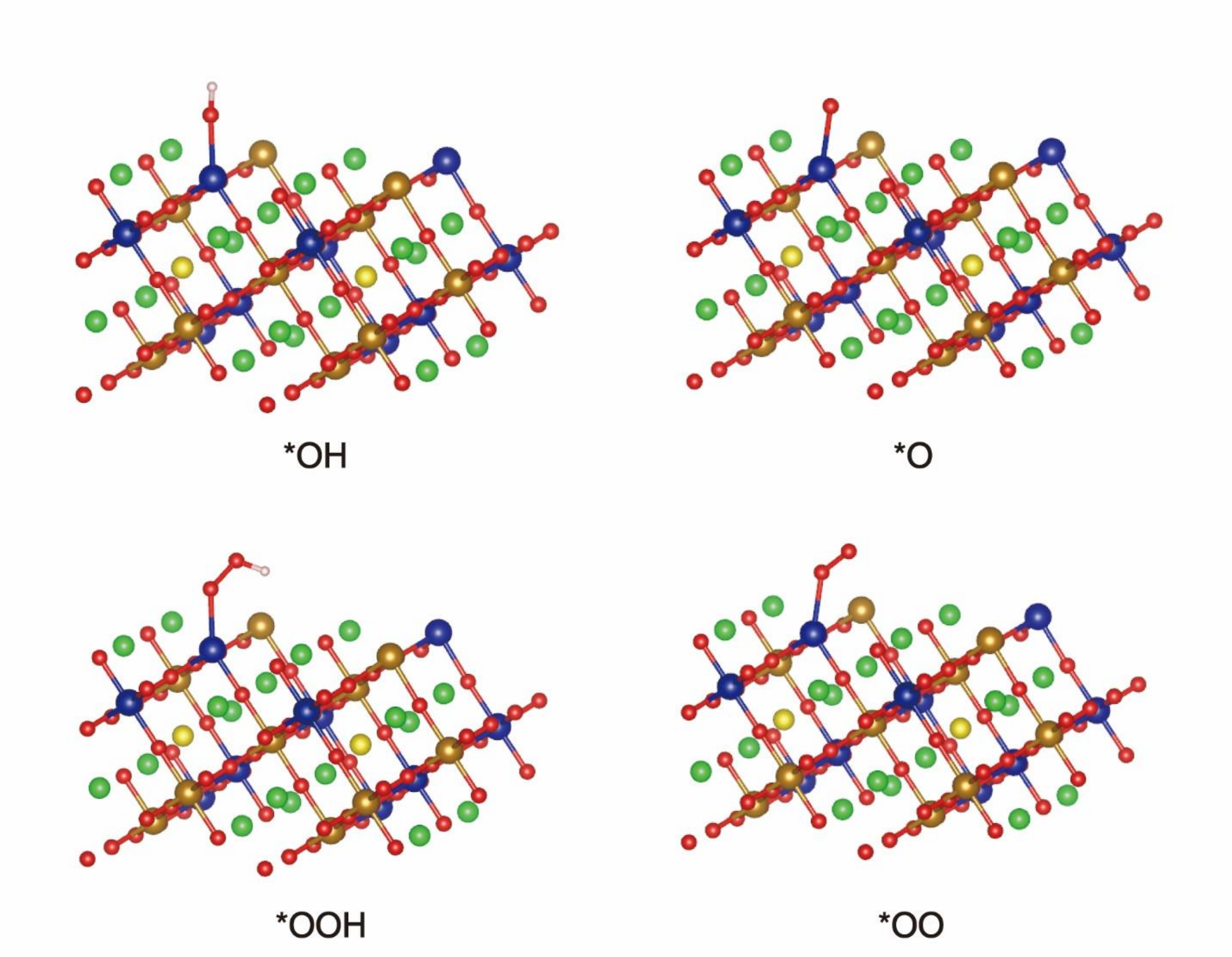

Supplementary Fig. 33. Side view of optimized configurations for OER pathway on PSCF via AEM. Brown, blue, red, pink, green, purple and yellow spheres represent Fe, Co, O, H, Sr, Mn and Pr element, respectively.

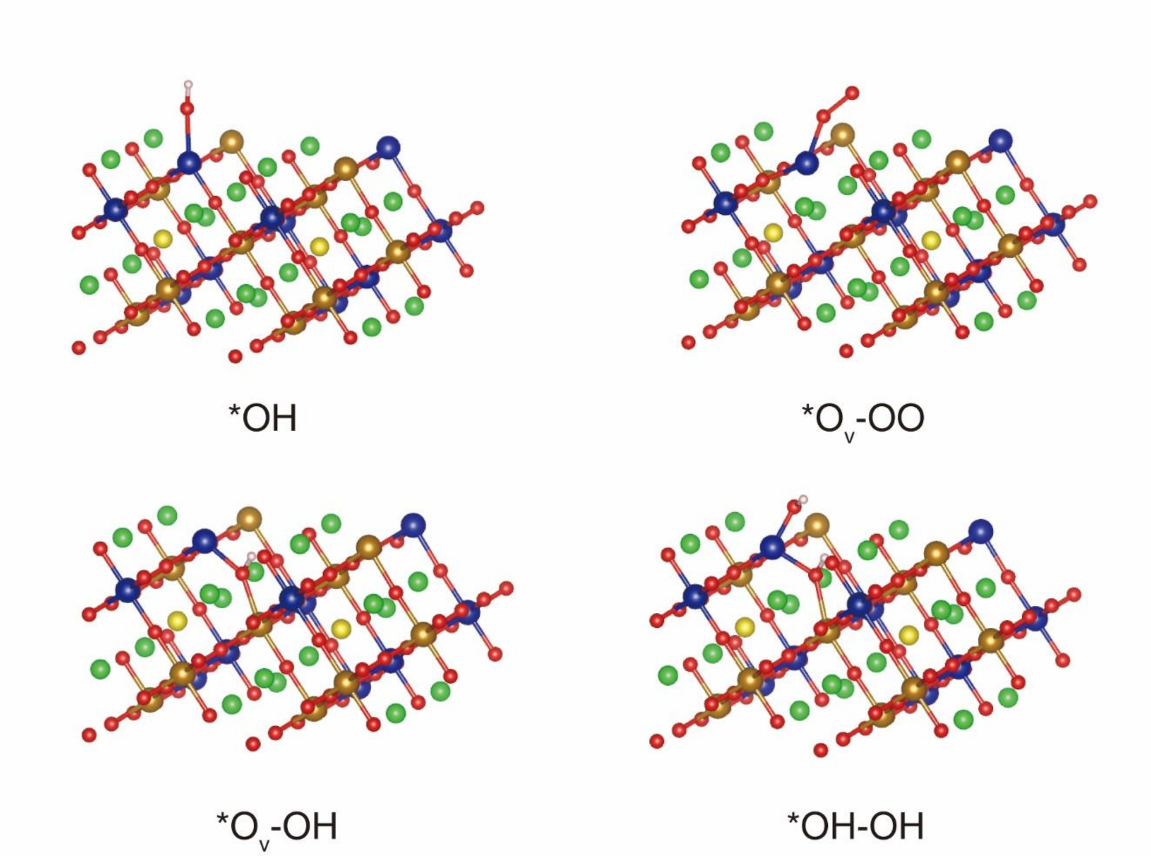

Supplementary Fig. 34. Side view of optimized configurations for OER pathway on PSCF via LOM. Brown, blue, red, pink, green, purple and yellow spheres represent Fe, Co, O, H, Sr, Mn and Pr element, respectively.

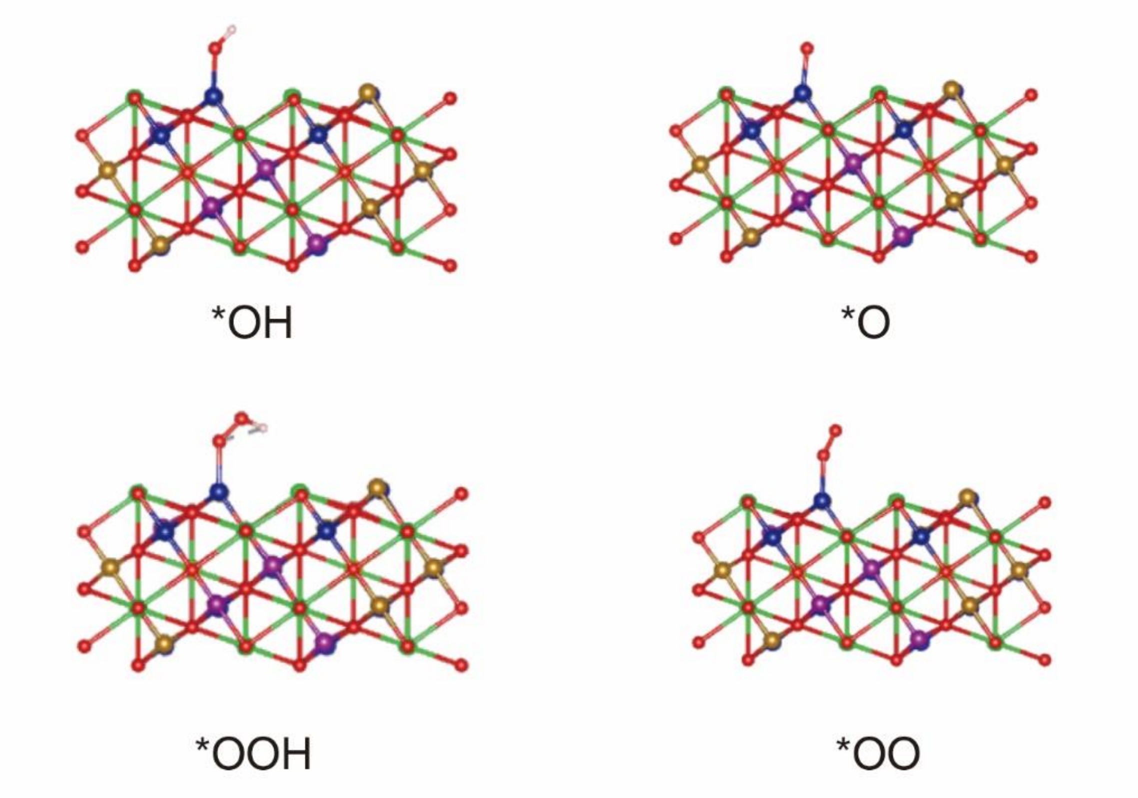

Supplementary Fig. 35. Side view of optimized configurations for OER pathway on PSCFM pathway via AEM. Brown, blue, red, pink, green, purple and yellow spheres represent Fe, Co, O, H, Sr, Mn and Pr element, respectively.

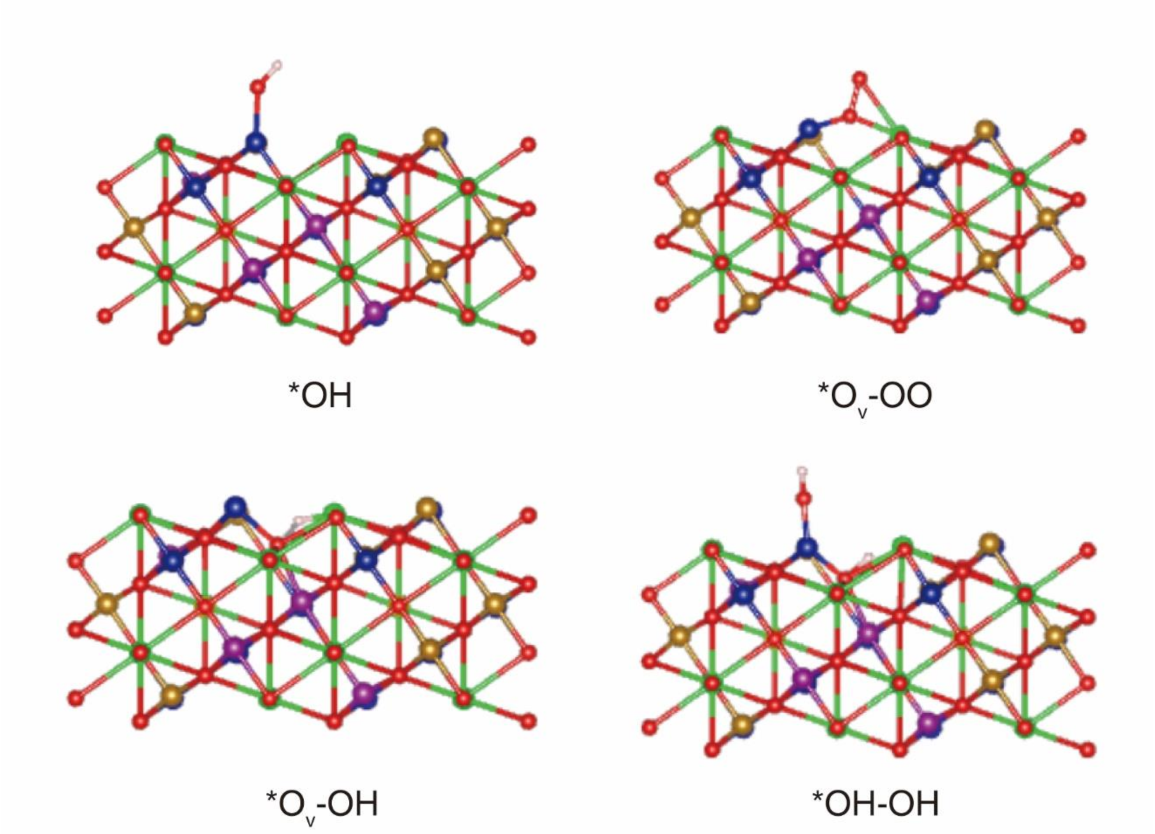

Supplementary Fig. 36. Side view of optimized configurations for OER pathway on PSCFM via LOM. Brown, blue, red, pink, green, purple and yellow spheres represent Fe, Co, O, H, Sr, Mn and Pr element, respectively.

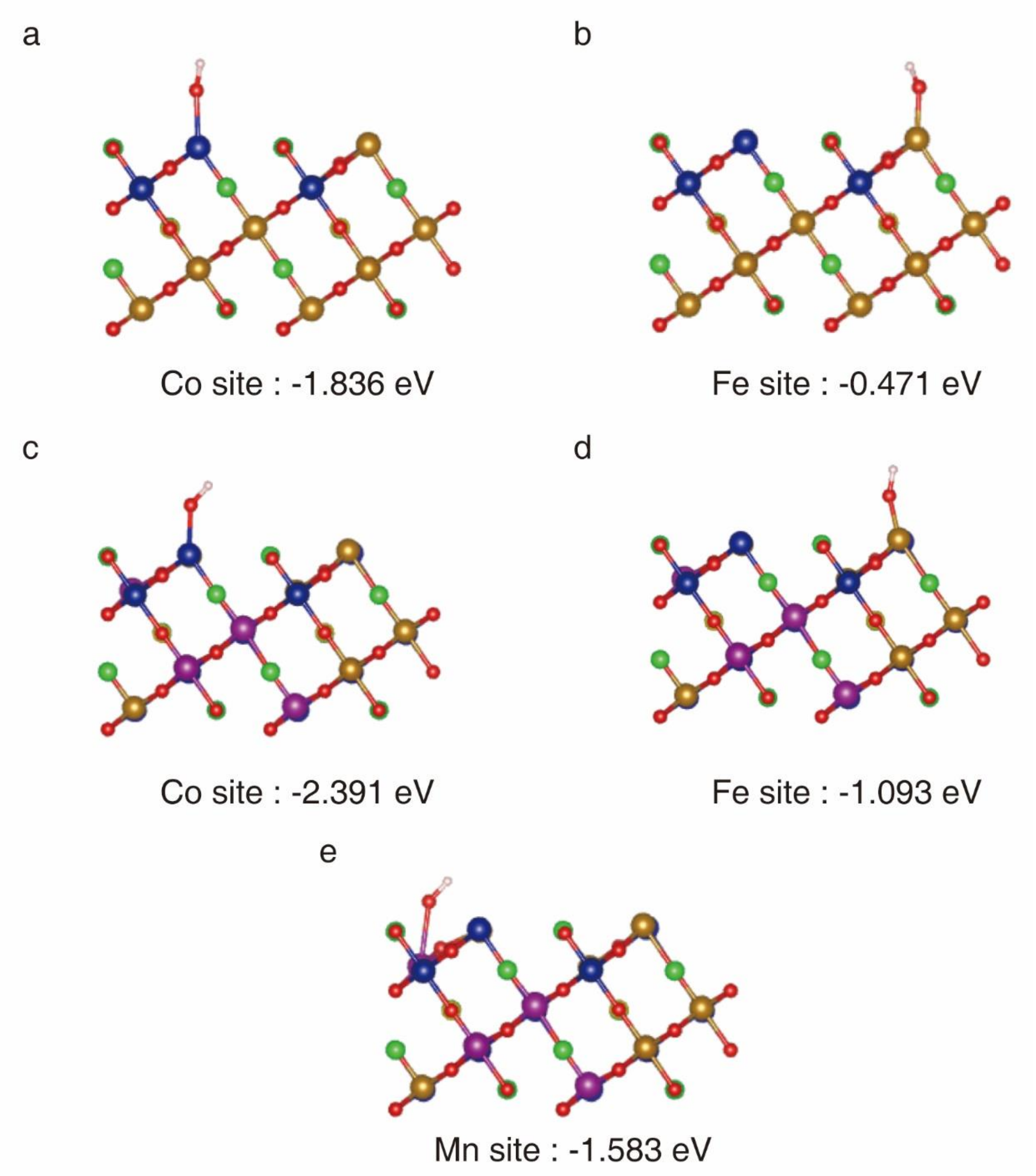

Supplementary Fig. 37. Side view of optimized configurations for OH absorption on (a) Co site and (b) Fe site in PSCF. Side view of optimized configurations for OH absorption on (c) Co site, (d) Fe site and (e) Mn site in PSCFM. Brown, blue, red, pink, green, purple and yellow spheres represent Fe, Co, O, H, Sr, Mn and Pr element, respectively.

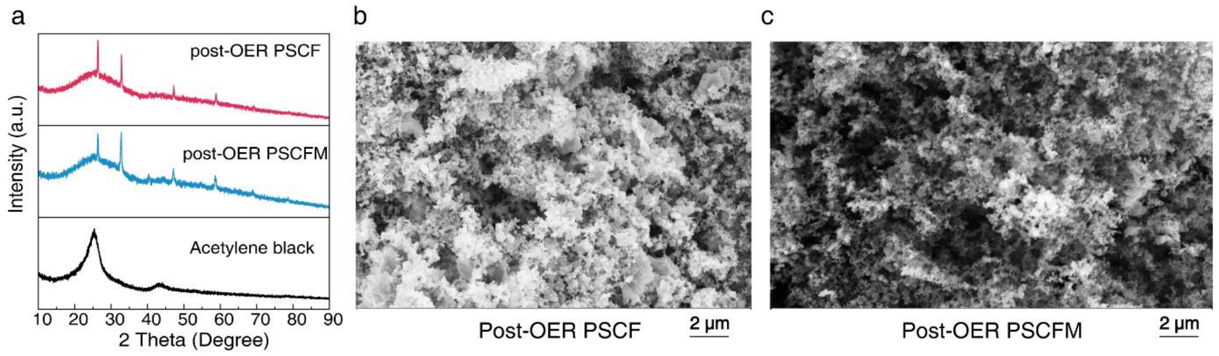

Supplementary Fig. 38. XRD patterns and SEM images of post-OER electrocatalysts. (**a**) XRD pattern of post-OER electrocatalysts and acetylene black. SEM images of (**b**) PSCF and (**c**) PSCFM after OER measurement. Source data are provided as a Source Data file.

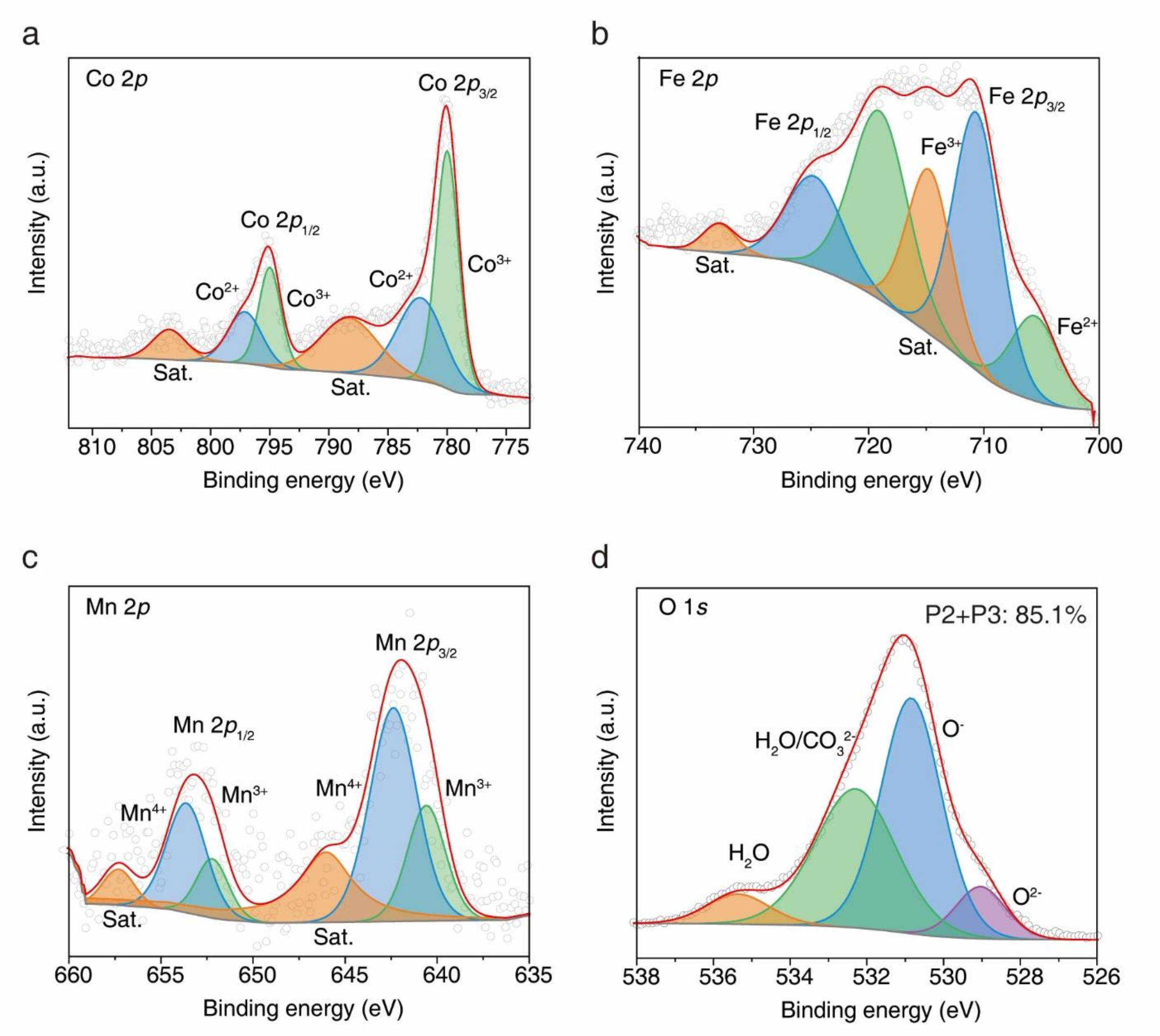

Supplementary Fig. 39. XPS spectra of spent PSCFM. The (**a**) Co 2*p*, (**b**) Fe 2*p*, (**c**) Mn 2*p* and (**d**) O 1*s* XPS spectrum of PSCFM after OER measurement. Source data are provided as a Source Data file.

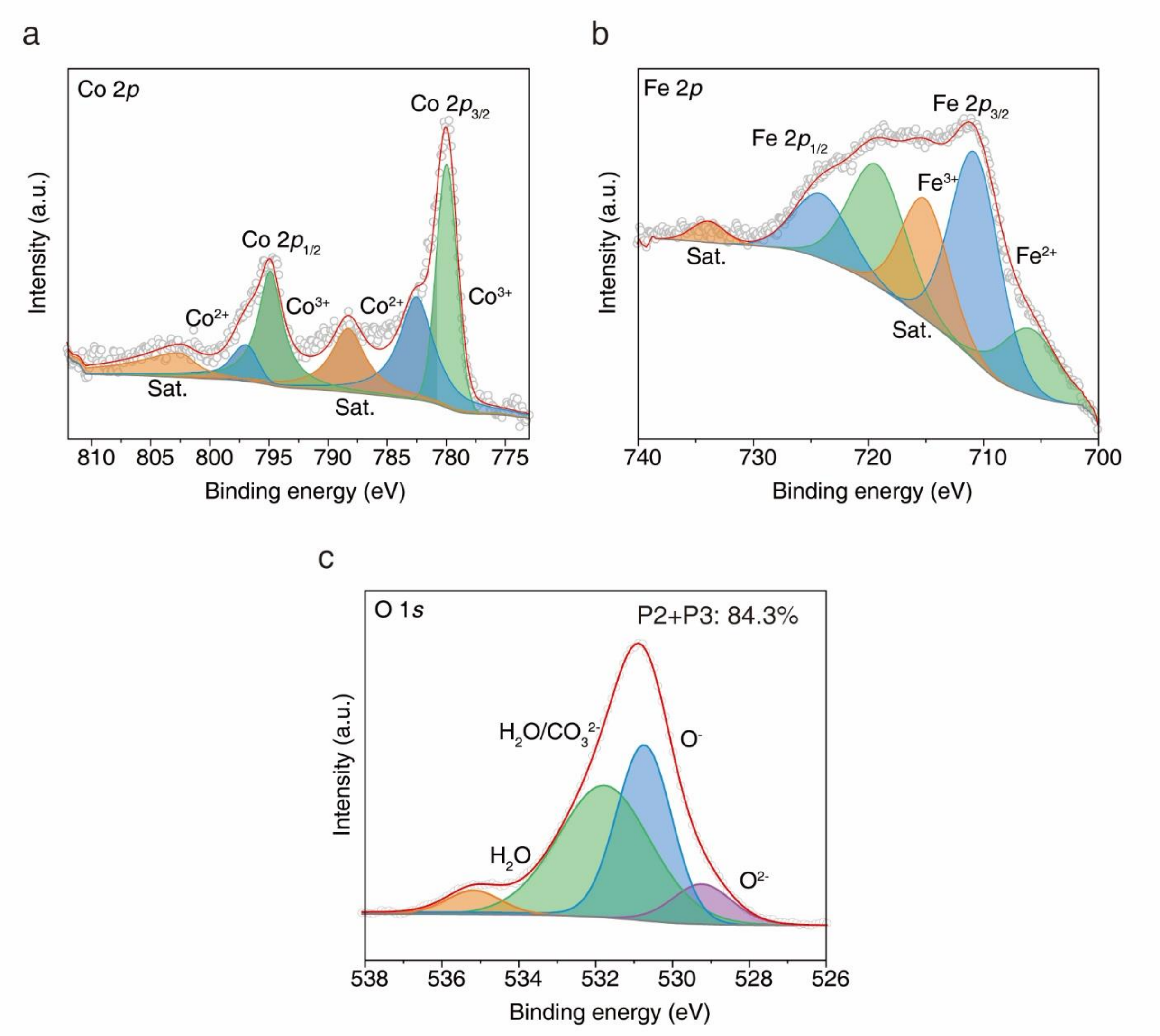

Supplementary Fig. 40. XPS spectra of spent PSCF. The (**a**) Co 2*p*, (**b**) Fe 2*p*, and (**c**) O 1*s* XPS spectrum of PSCF after OER measurement. Source data are provided as a Source Data file.

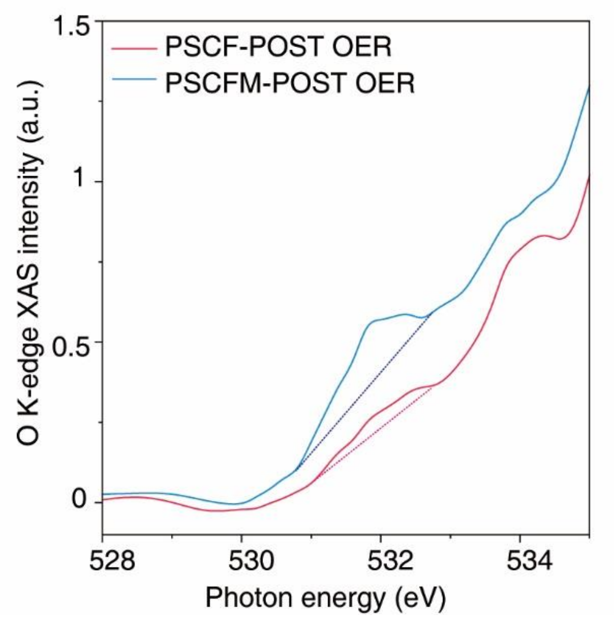

Supplementary Fig. 41. O K-edge XAS of PSCF and PSCFM after OER measurement.
Source data are provided as a Source Data file.

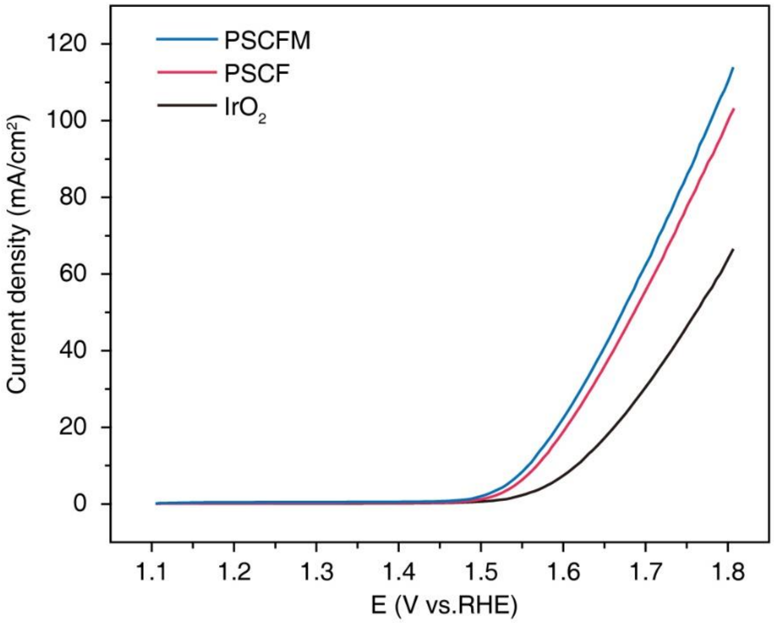

Supplementary Fig. 42. LSV curves of various electrocatalysts in 1 M KOH electrolyte without iR-correction. The test electrode area was 0.1963 cm^2. The R values for IrO_2, PSCF and PSCFM measurements were 5.98 ± 0.07, 6.0 ±0.04 and 6.0 ±0.06 Ω, respectively. Source data are provided as a Source Data file.

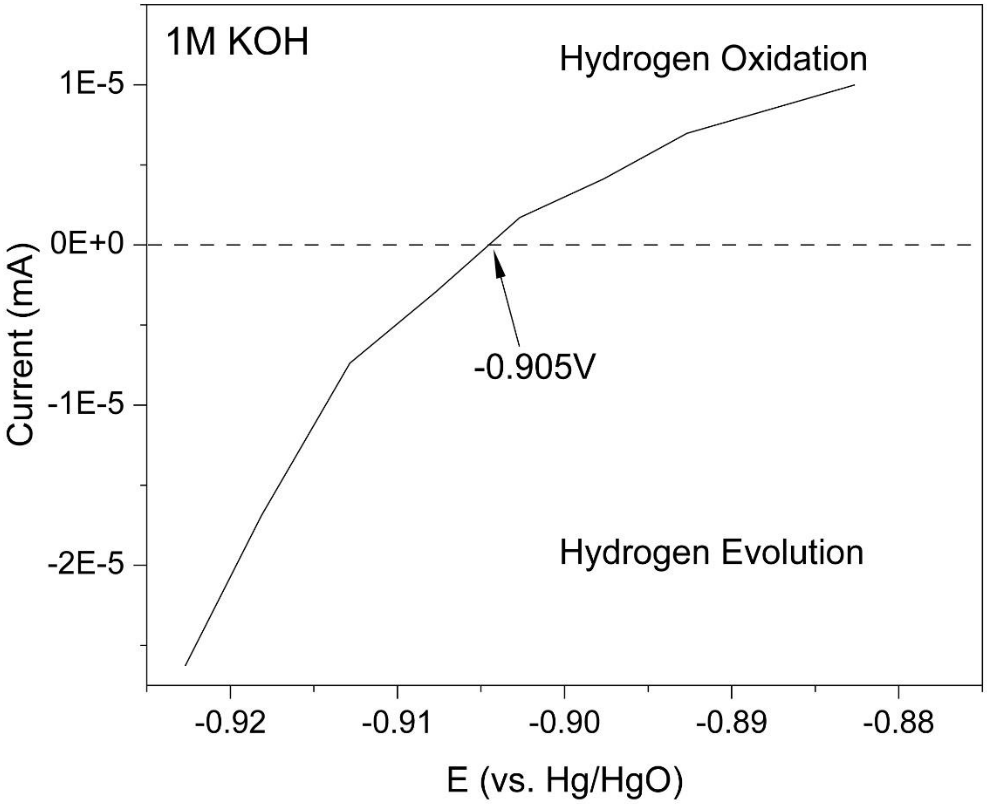

Supplementary Fig. 43. Calibration plot of Hg/HgO reference electrode. The potential of reversible hydrogen electrode is -0.905 V vs. Hg/HgO. Source data are provided as a Source Data file.

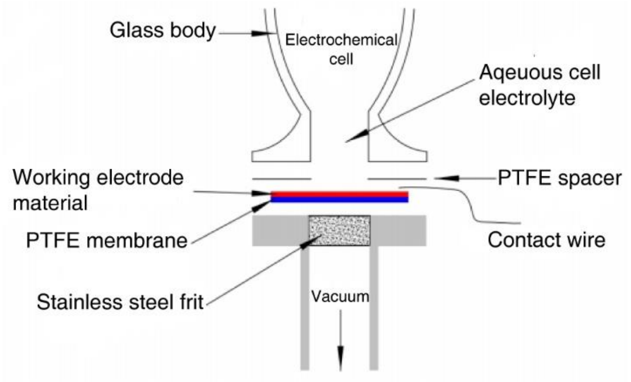

Supplementary Fig. 44. Schematic diagram of a classical DEMS electrochemical cell. The volume of liquid applied was quantified to be approximately 3 mL.

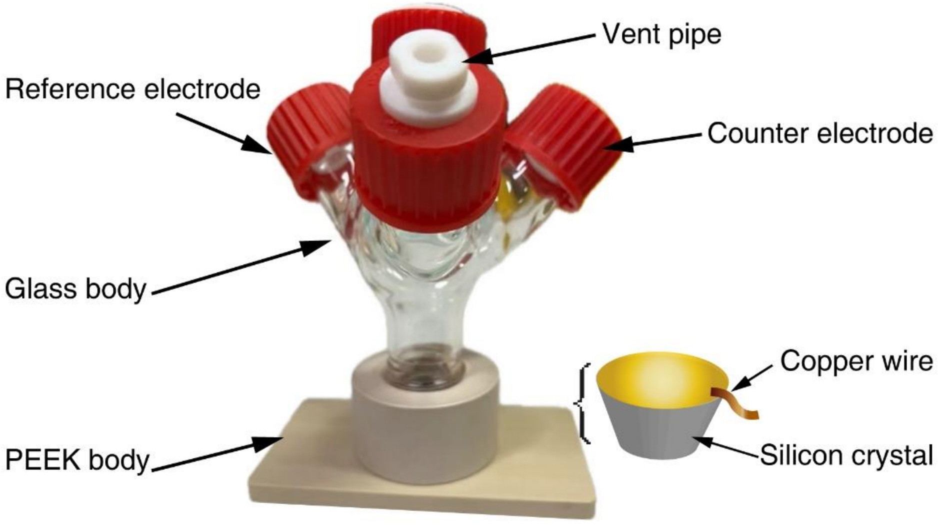

Supplementary Fig. 45. *In situ* infrared spectroscopy electrochemical cell. The volume of liquid applied was quantified to be approximately 10 mL. The apparatus utilized for *in situ* electrochemical attenuation total reflection Fourier transform infrared spectroscopy measurement can be found in elsewhere in: Infrared Phys. Technol., 2020, 105, 103200. Copyright 2020, Elsevier. [10]

Supplementary Table 1. A-Site cation electronegativity

Element	1+	2+	3+	4+	5+	6+
La			1.225			
Sr		1.093				
Ba		1.087				
Ca		1.092				
Pr			1.258			
Bi			1.434			
Sm			1.283			
Cs	0.984					
K	0.978					
Na	0.985					
Y			1.340			
Ce			1.348			
Sn				1.706		

Supplementary Table 2. B-Site cation electronegativity

Element	1+	2+	3+	4+	5+	6+
Mn*			1.627	1.912		
Cr			1.587			
Co*		1.377	1.693	2.009		
Cr			1.587			
Ni*		1.367	1.695	2.037		
V		1.545	1.795			
Fe*		1.390	1.651	1.912		
Mg		1.234				
Nb				1.69	1.862	
Ti				1.730		
Sn				1.706		
Ir				1.881		
Zr				1.610		
Y			1.340			
Ru				1.848		
Mo				1.808		2.101
Si				1.887		
Cu		1.372	1.9			
W						2.175

Elements marked with * are considered to coexist in different valence states during cation encoding.

Supplementary Table 3. First round prediction results

	Predicted overpotential (mV at 10 mA cm^{-2})			
Composition (AB)	Mean	Std	Min	Max
$Ba_{0.5}Sr_{0.5}Co_{0.7}Zr_{0.3}$	356.6	14.2	346.5	366.6
$Ba_{0.5}Sr_{0.5}Fe_{0.9}Sn_{0.1}$	378.4	15.9	367.2	389.6
$Ba_{0.7}Sr_{0.3}Co_{0.7}Mn_{0.3}$	337.7	7.2	335.7	378.3
$Ba_{0.7}Sr_{0.3}Co_{0.7}Zr_{0.3}$	385.1	13.0	375.9	394.3
$Ba_{0.7}Sr_{0.3}Fe_{0.9}Mn_{0.1}$	363.3	15.2	354.8	393.5
$Ba_{0.9}Sn_{0.1}Fe_{0.9}Ru_{0.1}$	349.0		349.0	349.0
$La_{0.1}Sr_{0.9}Sn_{0.9}Mn_{0.1}$	380.4		380.4	380.4
$La_{0.5}Sr_{0.5}Zr_{0.3}Ni_{0.7}$	394.8		394.8	394.8
$La_{0.7}Sr_{0.3}Fe_{0.5}Zr_{0.5}$	333.6	5.3	328.5	346.9
$La_{0.7}Sr_{0.3}Mn_{0.5}Cu_{0.5}$	384.9		384.9	384.9
$La_{0.7}Sr_{0.3}Mn_{0.7}Zr_{0.3}$	391.4		391.4	391.4
$La_{0.9}Ca_{0.1}Co_{0.5}Zr_{0.5}$	405.5	0.3	405.1	405.6
$La_{0.9}Sr_{0.1}Fe_{0.5}Ni_{0.5}$	400.5	22.5	353.8	428.5
$La_{0.9}Sr_{0.1}Mn_{0.3}Ni_{0.7}$	416.4		416.4	416.4
$La_{0.9}Sr_{0.1}Zr_{0.1}Ni_{0.9}$	411.8		411.8	411.8
$Pr_{0.1}Ba_{0.9}Co_{0.9}Zr_{0.1}$	360.3	2.3	356.0	362.7
$Pr_{0.1}Ba_{0.9}Fe$	377.2	28.7	350.0	434.5
$Pr_{0.1}Ba_{0.9}Fe_{0.9}Nb_{0.1}$	359.9	3.1	355.9	369.6
$Pr_{0.1}Ba_{0.9}Fe_{0.9}Ni_{0.1}$	360.0	3.5	355.6	384.1
$Pr_{0.1}Ba_{0.9}Fe_{0.9}Sn_{0.1}$	359.8	2.6	355.9	362.6
$Pr_{0.1}Sr_{0.9}Co_{0.3}Fe_{0.5}Ni_{0.2}$*	352.8	5.1	330.7	390.1
$Pr_{0.1}Sr_{0.9}Co_{0.5}Fe_{0.3}Mn_{0.2}$*	353.8	5.2	338.4	393.1
$Pr_{0.1}Sr_{0.9}Co_{0.5}Fe_{0.5}$	364.8	18.6	340.8	417.0
$Pr_{0.1}Sr_{0.9}Co_{0.5}Ru_{0.5}$	387.9	28.9	353.0	443.7
$Pr_{0.1}Sr_{0.9}Co_{0.9}Mn_{0.1}$	403.6	11.0	354.4	427.3
$Pr_{0.1}Sr_{0.9}Ni_{0.9}Ir_{0.1}$	410.1		410.1	410.1
$Pr_{0.1}Sr_{0.9}Ni_{0.9}Ru_{0.1}$	417.0		417.0	417.0
$Pr_{0.3}Ba_{0.7}Co_{0.3}Fe_{0.3}Cu_{0.4}$*	349.0	14.0	329.7	385.7
$Pr_{0.3}Ba_{0.7}Fe_{0.9}Mn_{0.1}$	373.7	4.2	372.2	398.9
$Pr_{0.3}Sr_{0.7}Co_{0.3}Fe_{0.5}Nb_{0.2}$*	361.4	10.1	347.7	399.5
$Pr_{0.5}Sr_{0.5}Co_{0.3}Fe_{0.3}Ni_{0.4}$*	354.6	6.4	344.4	365.8
$Pr_{0.5}Sr_{0.5}Co_{0.5}Fe_{0.3}Zr_{0.2}$*	360.9	14.1	330.5	401.1
$Pr_{0.7}Ca_{0.3}Co_{0.5}Zr_{0.5}$	431.5	62.8	327.5	478.5
$Pr_{0.7}Sr_{0.3}Co_{0.5}Sn_{0.5}$	445.9	20.0	416.8	476.9
$Pr_{0.9}Ca_{0.1}Co_{0.9}Cu_{0.1}$	378.7	20.4	367.7	500.3
$Pr_{0.9}Ca_{0.1}Fe_{0.5}Zr_{0.5}$	402.9	1.1	402.3	405.3

Compositions marked with * were predicted in the second round during the actual active learning cycle, and the predictive values of PSCF and PSCFM were added subsequently to validate the effectiveness of active learning. The absence of a standard deviation (Std) value signifies that the estimated valence state distribution is constrained to a single possible point (same below).

Supplementary Table 4. Second round prediction results

	Predicted overpotential (mV at 10 mA cm^{-2})			
Composition (AB)	Mean	Std	Min	Max
$Ba_{0.5}Sr_{0.5}Co_{0.7}Zr_{0.3}$	346.3	28.4	326.2	366.4
$Ba_{0.5}Sr_{0.5}Fe_{0.9}Sn_{0.1}$	336.3	20.5	321.8	350.8
$Ba_{0.7}Sr_{0.3}Co_{0.7}Mn_{0.3}$	334.2	27.4	301.7	370.6
$Ba_{0.7}Sr_{0.3}Co_{0.7}Zr_{0.3}$	386.2	4.8	382.9	389.6
$Ba_{0.7}Sr_{0.3}Fe_{0.9}Mn_{0.1}$	334.2	5.1	327.2	357.3
$Ba_{0.9}Sn_{0.1}Fe_{0.9}Ru_{0.1}$	321.6		321.6	321.6
$La_{0.1}Sr_{0.9}Sn_{0.9}Mn_{0.1}$	378.3		378.3	378.3
$La_{0.5}Sr_{0.5}Zr_{0.3}Ni_{0.7}$	330.1		330.1	330.1
$La_{0.7}Sr_{0.3}Fe_{0.5}Zr_{0.5}$	374.8	17.5	345.1	390.3
$La_{0.7}Sr_{0.3}Mn_{0.5}Cu_{0.5}$	362.6		362.6	362.6
$La_{0.7}Sr_{0.3}Mn_{0.7}Zr_{0.3}$	366.5		366.5	366.5
$La_{0.9}Ca_{0.1}Co_{0.5}Zr_{0.5}$	393.6	5.9	385.7	401.6
$La_{0.9}Sr_{0.1}Fe_{0.5}Ni_{0.5}$	390.6	13.3	358.4	421.9
$La_{0.9}Sr_{0.1}Mn_{0.3}Ni_{0.7}$	397.4		397.4	397.4
$La_{0.9}Sr_{0.1}Zr_{0.1}Ni_{0.9}$	378.3		378.3	378.3
$Pr_{0.1}Ba_{0.9}Co_{0.9}Zr_{0.1}$	395.4	4.8	387.3	402.2
$Pr_{0.1}Ba_{0.9}Fe$	350.1	33.9	320.1	439.3
$Pr_{0.1}Ba_{0.9}Fe_{0.9}Nb_{0.1}$	388.6	11.3	322.6	396.7
$Pr_{0.1}Ba_{0.9}Fe_{0.9}Ni_{0.1}$	382.4	18.6	321.5	406.9
$Pr_{0.1}Ba_{0.9}Fe_{0.9}Sn_{0.1}$	390.3	3.3	384.8	395.8
$Pr_{0.1}Sr_{0.9}Co_{0.3}Fe_{0.5}Ni_{0.2}$	320.2	12.8	302.9	398.3
$Pr_{0.1}Sr_{0.9}Co_{0.5}Fe_{0.3}Mn_{0.2}$	322.7	14.1	302.9	405.7
$Pr_{0.1}Sr_{0.9}Co_{0.5}Fe_{0.5}$	340.7	26.0	313.0	431.2
$Pr_{0.1}Sr_{0.9}Co_{0.5}Ru_{0.5}$	396.0	30.0	351.9	443.2
$Pr_{0.1}Sr_{0.9}Co_{0.9}Mn_{0.1}$	412.6	17.1	371.3	434.0
$Pr_{0.1}Sr_{0.9}Ni_{0.9}Ir_{0.1}$	348.7		348.7	348.7
$Pr_{0.1}Sr_{0.9}Ni_{0.9}Ru_{0.1}$	414.5		414.5	414.5
$Pr_{0.3}Ba_{0.7}Co_{0.3}Fe_{0.3}Cu_{0.4}$	362.6	33.3	301.5	412.0
$Pr_{0.3}Ba_{0.7}Fe_{0.9}Mn_{0.1}$	408.4	4.8	401.3	414.2
$Pr_{0.3}Sr_{0.7}Co_{0.3}Fe_{0.5}Nb_{0.2}$	377.2	14.5	337.1	406.4
$Pr_{0.5}Sr_{0.5}Co_{0.3}Fe_{0.3}Ni_{0.4}$	364.8	18.2	340.2	401.5
$Pr_{0.5}Sr_{0.5}Co_{0.5}Fe_{0.3}Zr_{0.2}$	363.3	12.4	324.7	388.7
$Pr_{0.7}Ca_{0.3}Co_{0.5}Zr_{0.5}$	365.4	23.6	336.9	406.1
$Pr_{0.7}Sr_{0.3}Co_{0.5}Sn_{0.5}$	389.1	23.3	350.2	434.1
$Pr_{0.9}Ca_{0.1}Co_{0.9}Cu_{0.1}$	384.1	6.5	375.1	413.0
$Pr_{0.9}Ca_{0.1}Fe_{0.5}Zr_{0.5}$	412.1	5.8	401.3	418.8

Supplementary Table 5. Third round prediction results

	Predicted overpotential (mV at 10 mA cm^{-2})			
Composition (AB)	Mean	Std	Min	Max
$Ba_{0.5}Sr_{0.5}Co_{0.7}Zr_{0.3}$	338.5	19.6	324.7	352.4
$Ba_{0.5}Sr_{0.5}Fe_{0.9}Sn_{0.1}$	349.6	26.7	330.7	368.4
$Ba_{0.7}Sr_{0.3}Co_{0.7}Mn_{0.3}$	344.7	10.7	332.3	409.9
$Ba_{0.7}Sr_{0.3}Co_{0.7}Zr_{0.3}$	344.9	43.9	313.8	376.0
$Ba_{0.7}Sr_{0.3}Fe_{0.9}Mn_{0.1}$	336.1	7.1	326.2	371.7
$Ba_{0.9}Sn_{0.1}Fe_{0.9}Ru_{0.1}$	327.4		327.4	327.4
$La_{0.1}Sr_{0.9}Sn_{0.9}Mn_{0.1}$	367.3		367.3	367.3
$La_{0.5}Sr_{0.5}Zr_{0.3}Ni_{0.7}$	421.4		421.4	421.4
$La_{0.7}Sr_{0.3}Fe_{0.5}Zr_{0.5}$	432.7	9.4	416.3	442.2
$La_{0.7}Sr_{0.3}Mn_{0.5}Cu_{0.5}$	393.6		393.6	393.6
$La_{0.7}Sr_{0.3}Mn_{0.7}Zr_{0.3}$	386.9		386.9	386.9
$La_{0.9}Ca_{0.1}Co_{0.5}Zr_{0.5}$	417.5	3.3	414.7	421.6
$La_{0.9}Sr_{0.1}Fe_{0.5}Ni_{0.5}$	416.3	14.6	379.1	443.1
$La_{0.9}Sr_{0.1}Mn_{0.3}Ni_{0.7}$	400.3		400.3	400.3
$La_{0.9}Sr_{0.1}Zr_{0.1}Ni_{0.9}$	414.6		414.6	414.6
$Pr_{0.1}Ba_{0.9}Co_{0.9}Zr_{0.1}$	330.8	3.8	323.8	335.0
$Pr_{0.1}Ba_{0.9}Fe$	346.0	41.1	309.8	432.2
$Pr_{0.1}Ba_{0.9}Fe_{0.9}Nb_{0.1}$	331.3	1.8	322.5	334.1
$Pr_{0.1}Ba_{0.9}Fe_{0.9}Ni_{0.1}$	332.1	6.1	320.8	370.1
$Pr_{0.1}Ba_{0.9}Fe_{0.9}Sn_{0.1}$	331.6	1.1	330.6	334.0
$Pr_{0.1}Sr_{0.9}Co_{0.3}Fe_{0.5}Ni_{0.2}$	326.2	9.7	313.6	400.9
$Pr_{0.1}Sr_{0.9}Co_{0.5}Fe_{0.3}Mn_{0.2}$	324.7	10.7	313.2	402.1
$Pr_{0.1}Sr_{0.9}Co_{0.5}Fe_{0.5}$	330.3	21.9	313.5	430.6
$Pr_{0.1}Sr_{0.9}Co_{0.5}Ru_{0.5}$	395.2	20.9	342.8	423.6
$Pr_{0.1}Sr_{0.9}Co_{0.9}Mn_{0.1}$	328.2	19.1	313.7	439.5
$Pr_{0.1}Sr_{0.9}Ni_{0.9}Ir_{0.1}$	317.8		317.8	317.8
$Pr_{0.1}Sr_{0.9}Ni_{0.9}Ru_{0.1}$	319.3		319.3	319.3
$Pr_{0.3}Ba_{0.7}Co_{0.3}Fe_{0.3}Cu_{0.4}$	364.8	44.0	316.7	417.4
$Pr_{0.3}Ba_{0.7}Fe_{0.9}Mn_{0.1}$	400.7	11.6	330.5	403.8
$Pr_{0.3}Sr_{0.7}Co_{0.3}Fe_{0.5}Nb_{0.2}$	396.8	13.1	348.9	435.4
$Pr_{0.5}Sr_{0.5}Co_{0.3}Fe_{0.3}Ni_{0.4}$	380.8	20.9	356.2	415.0
$Pr_{0.5}Sr_{0.5}Co_{0.5}Fe_{0.3}Zr_{0.2}$	353.7	15.8	306.3	407.1
$Pr_{0.7}Ca_{0.3}Co_{0.5}Zr_{0.5}$	436.4	26.7	381.1	459.4
$Pr_{0.7}Sr_{0.3}Co_{0.5}Sn_{0.5}$	452.4	16.1	425.8	469.1
$Pr_{0.9}Ca_{0.1}Co_{0.9}Cu_{0.1}$	419.3	4.7	411.0	428.9
$Pr_{0.9}Ca_{0.1}Fe_{0.5}Zr_{0.5}$	421.0	0.6	420.4	421.8

Supplementary Table 6. Predicted overpotential values for perovskite electrocatalysts based on machine learning results

Perovskite composition (ABO_3)	Overpotential (mV at 10 mA cm^{-2})
$Pr_{0.1}Sr_{0.9}Co_{0.5}Fe_{0.5}O_3$	326.9
$Pr_{0.1}Sr_{0.9}Co_{0.5}Ru_{0.5}O_3$	349.1
$Pr_{0.1}Sr_{0.9}Co_{0.9}Mn_{0.1}O_3$	444.4
$Pr_{0.5}Sr_{0.5}Co_{0.3}Fe_{0.3}Ni_{0.4}O_3$	352.2
$Pr_{0.3}Sr_{0.7}Co_{0.3}Fe_{0.5}Nb_{0.2}O_3$	396.1
$Pr_{0.1}Sr_{0.9}Co_{0.5}Fe_{0.3}Mn_{0.2}O_3$	314.8
$Pr_{0.1}Ba_{0.9}FeO_3$	399.4
$Pr_{0.1}Ba_{0.9}Fe_{0.9}Nb_{0.1}O_3$	414.6
$Pr_{0.9}Ca_{0.1}Co_{0.9}Cu_{0.1}O_3$	369.2
$La_{0.7}Sr_{0.3}Mn_{0.5}Cu_{0.5}O_3$	425.0
$Ba_{0.7}Sr_{0.3}Fe_{0.9}Mn_{0.1}O_3$	528.9
$Ba_{0.5}Sr_{0.5}Co_{0.7}Zr_{0.3}O_3$	416.0
$La_{0.7}Sr_{0.3}Fe_{0.5}Zr_{0.5}O_3$	N/A[a]

[a] $La_{0.7}Sr_{0.3}Fe_{0.5}Zr_{0.5}O_3$ exhibits poor OER activity, failing to reach a current density of 10 mA cm^{-2} in the LSV scan.

Supplementary Table 7. Calculated ECSA of various electrocatalysts

Sample	ECSA (cm^2)
PSCF	111.25
PSCFM	131.00
IrO_2	158.25

Supplementary Table 8. The fitting results of in-situ EIS measurement of various electrocatalysts

PSCF							
Potential (V vs. RHE)	R_s (Ω)	R_1 (Ω)	T_1 (S $s^{\alpha 1}$)	R_{ct} (Ω)	T_2 (S $s^{\alpha 2}$)	α1	α2
1.3	6.246	1.8747	0.0097924	89545	0.00025789	0.38	0.88
1.4	6.814	1.214	0.013014	43723	0.00038364	0.41	0.90
1.5	6.921	1.6433	0.010224	414.7	0.00048292	0.44	0.91
1.6	6.06	1.3341	0.047072	40.17	0.00064125	0.24	0.81
1.7	6.861	1.3044	0.010564	12.43	0.0027362	0.44	0.57
PSCFM							
Potential (V vs. RHE)	R_s (Ω)	R_1 (Ω)	T_1 (S $s^{\alpha 1}$)	R_{ct} (Ω)	T_2 (S $s^{\alpha 2}$)	α1	α2
1.3	6.345	1.135	0.0019141	62685	0.001194	0.79	0.93
1.4	6.621	1.951	0.0040394	22554	0.0021924	0.75	0.92
1.5	6.317	1.744	0.09594	84.94	0.0033408	0.33	0.87
1.6	6.501	0.9443	0.016214	8.893	0.0041504	0.80	0.79
1.7	6.517	1.3826	0.061694	2.881	0.0055002	0.40	0.72

Supplementary Table 9. The fitting results of chemical valences of various electrocatalyst based on XPS

Samples	Co (2+)	Co (3+)	Ave (Co)	Fe (2+)	Fe (3+)	Ave (Fe)	Mn (3+)	Mn (4+)	Ave (Mn)
PSCF	50.80%	49.92%	2.49	61.98%	38.40%	2.38	-	-	-
PSCFM	53.40%	46.60%	2.46	65.70%	34.30%	2.34	60.95%	39.05%	3.29
PSCF Post-OER	40.93%	59.07%	2.59	45.36%	54.64%	2.54	-	-	-
PSCFM Post-OER	52.55%	47.45%	2.47	54.81%	45.19%	2.45	30.44%	69.56%	3.69

Supplementary Table 10. Faraday efficiency of PSCF and PSCFM.

	1 min	5 min	10 min	30 min	1 h
PSCF	99.89%	96.03%	99.12%	98.25%	99.69%
PSCFM	98.50%	99.78%	97.73%	98.71%	99.05%

References

1. Ong, S. P. et al. Python Materials Genomics (pymatgen): A robust, open-source python library for materials analysis. *Comp. Mater. Sci.* **68,** 314-319 (2013).
2. Li, K. & Xue, D. Estimation of electronegativity values of elements in different valence states. *J. Phys. Chem. A* **110,** 11332-11337 (2006).
3. Ba, J. L., Kiros, J. R. & Hinton, G. E. Layer normalization. *arXiv preprint arXiv:1607.06450* (2016).
4. Kingma, D. P. & Ba, J. Adam: A method for stochastic optimization. arXiv Preprint at https://arxiv.org/abs/1412.6980. (2014).
5. Friedman, J. H. Greedy function approximation: a gradient boosting machine. *Ann. Stat.* **29,** 1189-1232 (2001).
6. Ostrovsky, R., Rabani, Y., Schulman, L. J. & Swamy, C. The effectiveness of lloyd-type methods for the K-means problem. *J. Acm* **59,** 1-22 (2013).
7. Chacón, J. E. & Rastrojo, A. I. Minimum adjusted Rand index for two clusterings of a given size. *Adv. Data Anal. Classi.* **17,** 125-133 (2022).
8. Rosenberg, A. & Hirschberg, J. V-measure: a conditional entropy-based external cluster evaluation measure. *In Proc. 2007 Joint Conference on Empirical Methods in Natural Language Processing and Computational Natural Language Learning (EMNLP-CoNLL)* 410-420 (Association for Computational Linguistics, 2007).
9. Rousseeuw, P. J. Silhouettes: a graphical aid to the interpretation and validation of cluster analysis. *J. Comput. Appl. Math.* **20,** 53-65 (1987).
10. Hu, C., Wang, X., Qi, Z. & Li, C. The new infrared beamline at NSRL. *Infrared Phys. Technol.* **105,** 103200 (2020).
