# Supplementary material for: Transfer learning guided discovery of efficient perovskite oxide for alkaline water oxidation
Source: Nat Commun. 2024 Jul 26;15:6301. doi: 10.1038/s41467-024-50605-5 (PMC11282268; doi:10.1038/s41467-024-50605-5)
Supplement: Supplementary file 3 — Description of Additional Supplementary Files [file 41467_2024_50605_MOESM3_ESM.pdf]

Description of Additional Supplementary Files

Supplementary Data 1: Source data used for training ML models, including cation encoding and embedding data, prediction data for the first and second rounds (with third-round data provided in the main text). CIF data for the synthesized materials are also included.
